# Efficacy of acupuncture as adjunctive therapy for patients with acute exacerbation of chronic obstructive pulmonary disease: a systematic review and meta-analysis

**DOI:** 10.3389/fmed.2025.1513888

**Published:** 2025-05-12

**Authors:** Guofeng Li, Jimin Liu, Guixian Yang, Jiajia Li, Yuan He, Xinru Fei, Lai Wei, Dongkai Zhao

**Affiliations:** ^1^College of Traditional Chinese Medicine, Changchun University of Traditional Chinese Medicine, Changchun, China; ^2^The Third Affiliated Clinical Hospital of Changchun University of Chinese Medicine, Changchun, China; ^3^Changzhongda Traditional Diagnosis and Treatment Hospital in Jilin Province, Changchun, China

**Keywords:** acupuncture, acute exacerbation of chronic obstructive pulmonary disease, efficacy, systematic review, meta-analysis

## Abstract

**Background:**

Chronic obstructive pulmonary disease (COPD) is a highly prevalent and potentially fatal respiratory condition. Acute exacerbations can accelerate lung function decline and increase mortality. Acupuncture has been increasingly used as an adjunctive treatment for respiratory diseases, but its effectiveness in acute exacerbations of COPD (AECOPD) remains controversial. Existing evaluations on this topic are limited in scope and depth. This study aimed to provide a more comprehensive review to evaluate the effectiveness of acupuncture as an adjuvant treatment for acute exacerbations of chronic obstructive pulmonary disease.

**Study design:**

Systematic review and meta-analysis of existing randomized controlled trials on acupuncture-assisted treatment of acute exacerbation of chronic obstructive pulmonary disease (AECOPD).

**Methods:**

We included randomized controlled trials (RCTs) comparing acupuncture combined with conventional Western medicine to conventional Western medicine alone in patients with acute exacerbations of COPD (AECOPD). Our literature search covered ten databases, including PubMed and Web of Science ect., up until March 2025. The primary outcome was the effective rate, while secondary outcomes included lung function (FEV_1_%, FEV_1_/FVC%, FEV_1_), arterial blood gas analysis (PaO_2_, PaCO_2_, SaO_2_), the 6-min walk test (6MWT), COPD Assessment Test (CAT), modified Medical Research Council (mMRC) scale, and success rate of weaning. Data were extracted from eligible studies, and statistical analysis was performed using RevMan 5.3 and Stata 16.0. Risk of bias and evidence quality were assessed using Cochrane tools and GRADE methodology.

**Results:**

The study included 31 randomized controlled trials (RCTs) with 2,299 participants. The studies were primarily conducted in hospital inpatient departments, and the typical treatment duration ranged from 1 to 2 weeks. Compared with conventional Western medicine alone, acupuncture combined with conventional Western medicine showed greater effectiveness (RR = 1.23, 95%CI 1.17 ~ 1.29, *p* < 0.001). Acupuncture significantly improved lung function (FEV_1_%: MD = 5.67, 95%CI 2.97 ~ 8.37, *p* < 0.001; FEV_1_/FVC: MD = 4.44, 95%CI 1.86 ~ 7.03, *p* < 0.001; FEV_1_: MD = 0.37, 95%CI 0.26 ~ 0.47, *p* < 0.001), reduced hypoxia (PaO_2_: MD = 3.60, 95%CI 2.23 ~ 4.98, *p* < 0.001; PaCO_2_: MD = -3.30, 95%CI -5.80 ~ −0.80, *p* < 0.05; SaO_2_: MD = 4.23, 95%CI 3.02 ~ 5.43, *p* < 0.001), and improved exercise tolerance (6MWT: MD = 40.34, 95%CI 30.50 ~ 50.17, *p* < 0.001), quality of life (CAT: MD = -2.68, 95%CI -3.39 ~ −1.96, *p* < 0.001), and dyspnea (mMRC: MD = -0.33, 95%CI -0.47 ~ −0.20, *p* < 0.001). However, the weaning success rate did not show a statistically significant difference between the two groups (RR = 1.18, 95%CI 0.95 ~ 1.48, *p* = 0.14). Mild side effects were reported in some studies. We rated the quality of evidence as very low to medium.

**Conclusion:**

This systematic review and meta-analysis demonstrate that acupuncture, as an adjunctive treatment for acute exacerbations of chronic obstructive pulmonary disease, improves clinical efficacy and key outcomes. Our findings are consistent with previous studies that demonstrated improvements in the COPD Assessment Test (CAT) and arterial blood gas parameters (PaO2 and PaCO2). Unlike previous meta-analyses, the present study showed that adjunctive acupuncture significantly improved patient lung function FEV_1_% outcomes and significantly improved patient 6-min walk distance and modified Medical Research Council (mMRC) score; however, there was no significant difference in the success rate of weaning between the two groups. Although the review highlights clinical benefits, the heterogeneity of the included studies and the overall quality of the evidence suggest that more high-quality randomized controlled trials are needed to validate these findings and optimize treatment strategies. These studies should also prioritize standardizing acupuncture regimens, extending treatment duration, and conducting long-term follow-up assessments.

**Systematic Review Registration:**

https://www.crd.york.ac.uk/prospero/ ID:CRD42024528155.

## 1 Introduction

Chronic obstructive pulmonary disease (COPD) is a heterogeneous pulmonary condition, usually characterized by persistent, progressive and aggravated airflow obstruction (1), and has become a major global public health challenge due to its high morbidity and mortality (2). Acute attacks can lead to disease progression, and frequent attacks can accelerate the decline of lung function. After one acute moderate-to-severe exacerbation, the decline rate of lung function in patients increases by more than 95% (3), and the cardiopulmonary risk of patients is greatly increased and the mortality of patients is increased (4, 5). AECOPD is defined as the deterioration of respiratory symptoms within 14 days. It is closely related to the increase of local or systemic inflammatory response caused by respiratory infection and other factors (1). The current mainstream treatment is still corticosteroids and non-invasive ventilation, but the efficacy is limited (6). In recent years, more and more studies (7–10) have shown that integrated treatment of traditional Chinese and western medicine can better alleviate the symptoms of acute attacks of COPD patients, improve lung function, and improve the efficiency of clinical treatment.

Acupuncture, as an important part of the external treatment of traditional Chinese medicine, has been widely used in the clinical treatment of various pain diseases (11, 12) and nervous system diseases such as insomnia and headache (13, 14). It has been proved effective in anti-inflammation, immune regulation, and autonomic nervous system regulation. At present, acupuncture is widely used in the prevention and treatment of various chronic respiratory diseases (15). The World Health Organization has also listed diseases such as bronchitis and bronchial asthma as conditions for which acupuncture is effective (16), and the 2021 Global Initiative for Chronic Obstructive Pulmonary Disease (GOLD) update acknowledges that acupuncture can potentially improve breathing difficulties and quality of life in COPD patients (17). Studies have indicated that acupuncture can reduce systemic and airway inflammation (18), improve blood rheology (19), and enhance immune function (20), thereby improving small airway obstruction, reducing recurrent attacks, and improving exercise endurance.

In recent years, more and more studies have been conducted on the acupuncture-assisted treatment of AECOPD, but the results are inconsistent. For example, Yang et al. (21) added abdominal acupuncture treatment on the basis of conventional treatment, and the results indicated that PaO_2_ was significantly increased, PaCO_2_ was significantly decreased, and invasive ventilation time was significantly reduced compared with the conventional treatment group. Gui et al. (22) randomly divided 251 AECOPD patients into a control group and a study group. The study group received acupuncture treatment on the basis of the treatment of the control group, and compared with the control group, the inflammatory factors such as IL-4 and IL-18 were significantly lower in the study group, and the CAT and mMRC scores were also statistically significantly lower, which effectively improved the quality of life and degree of dyspnea. Due to the particularity of acupuncture treatment, the differences in acupuncture methods, acupuncture points, stimulation frequency, and whether acupuncture is used in different schemes may have a certain impact on the results, so the research results are also different. In the study of Oncu et al. (23), the addition of acupuncture did not improve lung function or the 6-min walking distance. Gao Y et al. (24) showed that although electroacupuncture could improve the clinical symptoms of patients with dyspnea, the experimental group had no obvious advantages compared with the control group in CAT score and BODE index. Therefore, a meta-analysis of acupuncture treatment for AECOPD is necessary to uncover its true efficacy.

In a recently published meta-analysis on acupuncture treatment of AECOPD (25), the scope of inclusion of acupuncture was small, with only 12 articles included. Considering the small scope of inclusion, the advantages of acupuncture treatment of AECOPD could not be reflected. Based on the reference of relevant literatures (26, 27), we expanded the scope of inclusion of acupuncture types. To comprehensively evaluate the efficacy and safety of acupuncture-assisted treatment of AECOPD, and to explore the possible mechanism, so as to provide evidence for clinical practice.

## 2 Materials and methods

This systematic review was conducted as per the Preferred Reporting Items for Systematic Reviews and Meta-Analyses (PRISMA) guideline (Supplementary material 1). This review has been registered on ROSPERO (ID: CRD42024528155), which is accessible at https://www.crd.york.ac.uk/PROSPERO/.

### 2.1 Inclusion criteria

(1) The articles included in our study were randomized controlled trials (RCTs). (2) Patients diagnosed with AECOPD according to the Global Initiative for Chronic Obstructive Lung Disease (GOLD) or Chinese COPD expert consensus and guidelines were included, regardless of age, gender, and race. (3) The experimental group was given acupuncture treatment on the basis of the control group. (4) Control group: The control group received conventional Western medicine treatment (antibiotics, corticosteroids, bronchodilators, etc.) or mechanical ventilation. (5) Primary outcome measures included the effective rate. Secondary outcome measures included lung function (including FEV_1_%, FEV_1_/FVC%, and FEV_1_), arterial blood gas analysis (including PaO_2_, PaCO_2_, and SaO_2_), 6-min walk test (6MWD), COPD Assessment Test (CAT), modified Medical Research Council (mMRC), and success rate of weaning. These measurements were carried out on at least one of these outcomes.

### 2.2 Exclusion criteria

(1) Animal studies, (2) Repeated publications, (3) The analytical data was either incomplete or omitted, rendering it impossible to extract. This could be due to the unavailability of the full text, key outcome indicators, or the inability to convert the data. (4) To explore the true efficacy of acupuncture more accurately, neither the experimental group nor the control group received other traditional Chinese medicine treatments or rehabilitation therapies, such as Chinese herbal medicine, cupping, moxibustion, acupoint application, acupoint embedding, acupoint injection, or extracorporeal counterpulsation, etc. (5) Focus on researching other organ complications and comorbidities related to AECOPD, such as insomnia, anxiety, pulmonary encephalopathy, malnutrition, and complications caused by mechanical ventilation such as abdominal distension.

### 2.3 Search strategy

The search was completed on March 2025, and the databases employed in the search were as follows: CNKI, Wanfang Data, China Biomedical Literature Database, PubMed, Chongqing VIP, Embase, Web of Science, Cochrane Library, AMED and CINAHL.

The MESH keywords used for searching the articles were carefully selected to ensure comprehensive coverage of the relevant literature. The primary MESH terms and their combinations included: “Pulmonary Disease, Chronic Obstructive” (COPD); “Symptom Flare Up”; “Inpatients”; “Acupuncture”; “Acupuncture Therapy.” These MESH terms were combined using Boolean operators to enhance the search strategy, for example: (“Pulmonary Disease, Chronic Obstructive” OR “COPD”) AND (“Symptom Flare Up”) AND (“Acupuncture” OR “Acupuncture Therapy”) (Supplementary material 2).

### 2.4 Data extraction

Standardized data extraction tables were developed prior to the review. The table includes fields such as study characteristics (author, year, country), population details (sample size, age, sex), intervention and control details, outcome measures, and study results.

After the completion of the literature search, the reviewers (JML and GXY) independently used the software EndNote X9 to reduce duplicates and conduct preliminary analyses of titles and abstracts. The final inclusion was determined by reading the complete text under the previously established inclusion and exclusion criteria. The opinions of a third reviewer (GFL) were obtained in case of inconsistency. All downloaded articles were examined, and the data was extracted *via* a predetermined table. The following details including first author, publication year, gender ratio, average age, treatment group, comparison group, sample size, acupuncture information, acupoint, and outcome measurement were all recorded. The calculated kappa value was *κ* = 0.798, indicating a substantial level of agreement between the reviewers.

### 2.5 Assess the risk of bias

The risk of bias in the included literature was evaluated by two reviewers (JJL and YH) who used the Cochrane bias tool from RCT (28). Five factors were analyzed: (1) selection bias (random sequence formation and assignment masking), (2) performance bias (blind participants and providers), (3) detection bias (outcome evaluators), (4) attrition bias (incomplete outcome data), and (5) reporting bias (selective outcome reporting). The analyses were categorized as low risk, high risk, and undetermined (29), and any queries were resolved *via* a discussion with the third reviewer (XRF). Lastly, the abovementioned five factors will summarize the overall risk of biased judgment.

### 2.6 Assess the quality of evidence

This study used the Graded Assessment, Development, and Evaluation (GRADE) system to evaluate the quality of the evidence in the included studies (30). High, medium, low, and very low were the four levels of evidence quality. The true effect was more closely aligned with the predicted value as the level of evidence increased, and five lowering factors (study constraints, uniformity of effects, inaccuracies, interrelatedness, and publication bias) were completely considered. After reviewing it by both (JML) and (LW) reviewers, the third reviewer (GFL) has reviewed the document again to cross-check any objections.

### 2.7 Statistical analysis

Data was statistically examined *via* ReMan 5.3 and Stata 16.0 software. The mean difference (MD) and its 95% CI were used to describe continuous variables. The influence of dichotomous data was analyzed using relative risk (RR) with a 95% CI. Based on the significance threshold of *α* = 0.05, any *p* ≤ 0.05 was deemed to be significant. When *p* > 0.1 and *I*^2^ < 50%, it indicates the absence of heterogeneity. The *p*-value ≤0.1 or the *I*^2^ ≥ 50%, represented the presence of heterogeneity. We conducted a sensitivity analysis by systematically excluding each study individually. Randomized or fixed-effect models were selected for meta-analysis after excluding clinical or methodological heterogeneity. When it was not possible to exclude it, the source of heterogeneity was further identified *via* subgroup analysis. When we included more than 10 studies, we assessed publication bias using a funnel plot. The symmetry of the funnel plot was tested *via* Egger’s test. If *p* ≥ 0.05 indicates that the funnel plot was symmetrical and there was no evidence of publication bias among included studies.

## 3 Results

### 3.1 Search analysis

**Figure 1:**
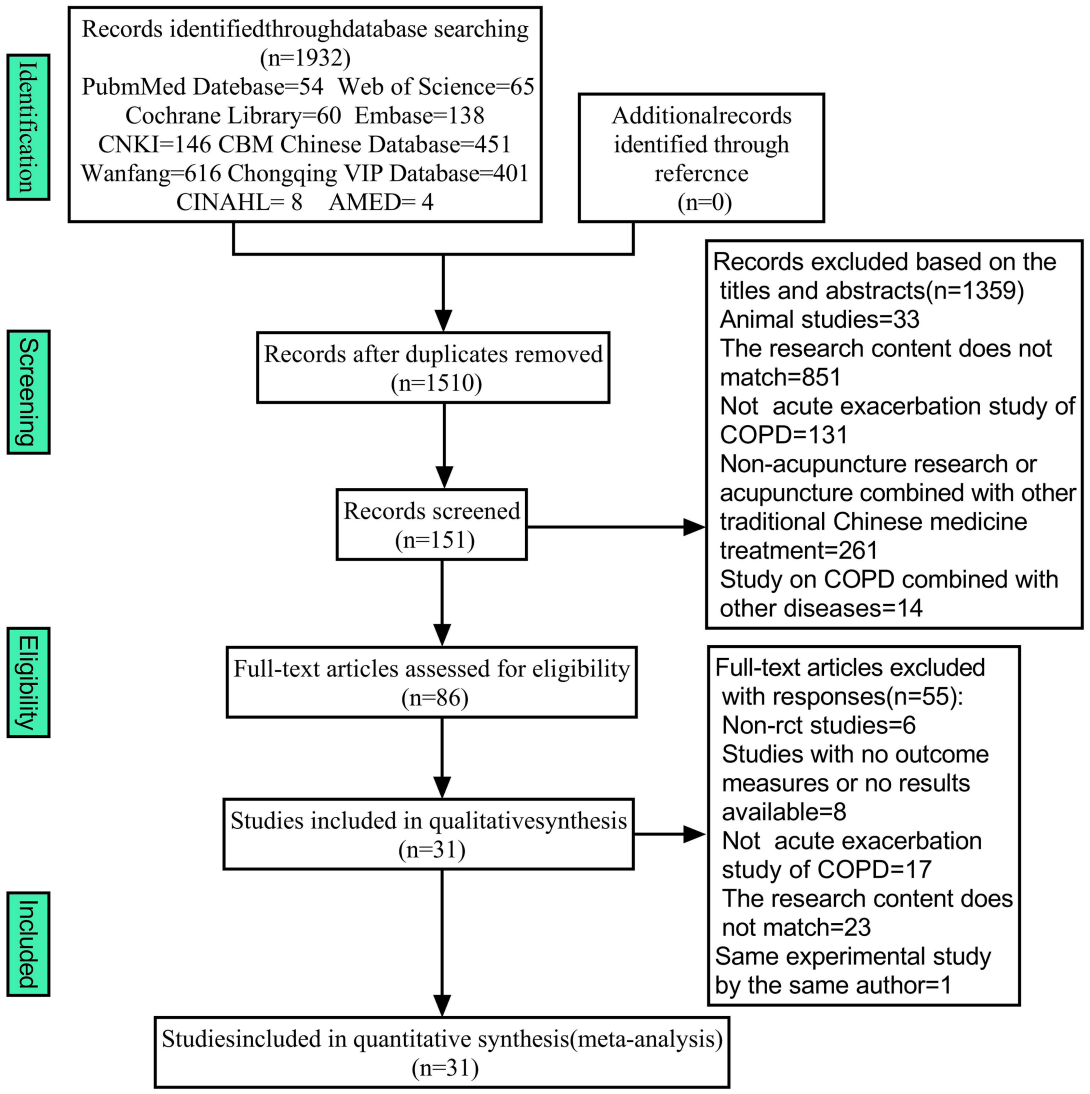
Flow chart of literature screening.

A total of 1932 articles were obtained from 10 databases. There were 1,510 articles remaining after the removal of duplicates. After reviewing the abstract and title, 1,359 articles were dropped due to their failure to satisfy the inclusion criteria. Almost 86 articles that satisfied the initial screening were re-screened by reviewing the full text. A total of 55 articles were excluded due to their failure to satisfy the inclusion criteria, and 31 RCTs were ultimately incorporated into the meta-analysis (Figure 1).

### 3.2 Features of the included study

This meta-analysis comprised 31 articles, 33 research papers, and a total of 2,299 participants. The sample sizes range from 40 to 251. Most of the studies were conducted in hospital settings in China, reflecting the prevalence of acupuncture in the region. The duration of the intervention ranged from 3 days to 4 weeks, and all studies assessed short-term treatment effects. The experimental group consisted of 1,175 individuals, whereas the control group had 1,124 individuals. The study population is mainly middle-aged and elderly, with more men than women. Among the studies, Gui et al. (22) had the highest number of cases with 251 patients, while Yuwen et al. (31) and Wen et al. (32) both had the lowest number of cases with 40 patients. Among 31 studies, 5 studies used abdominal needles, 3 studies used electroacupuncture, 1 studies used intradermal needles, 1 study used Fu’s acupuncture, 1 study used ear needles, 1 study used warm needles, and the remaining studies used traditional acupuncture. Both studies conducted by Guan et al. (33) and Cheng et al. (34) involved seven outcome indicators, which was the highest number of outcome indicators. In all of the experiments, feishuxue (BL13) was the most frequently selected (Supplementary material 3).

### 3.3 Risk of bias

**Figure 2:**
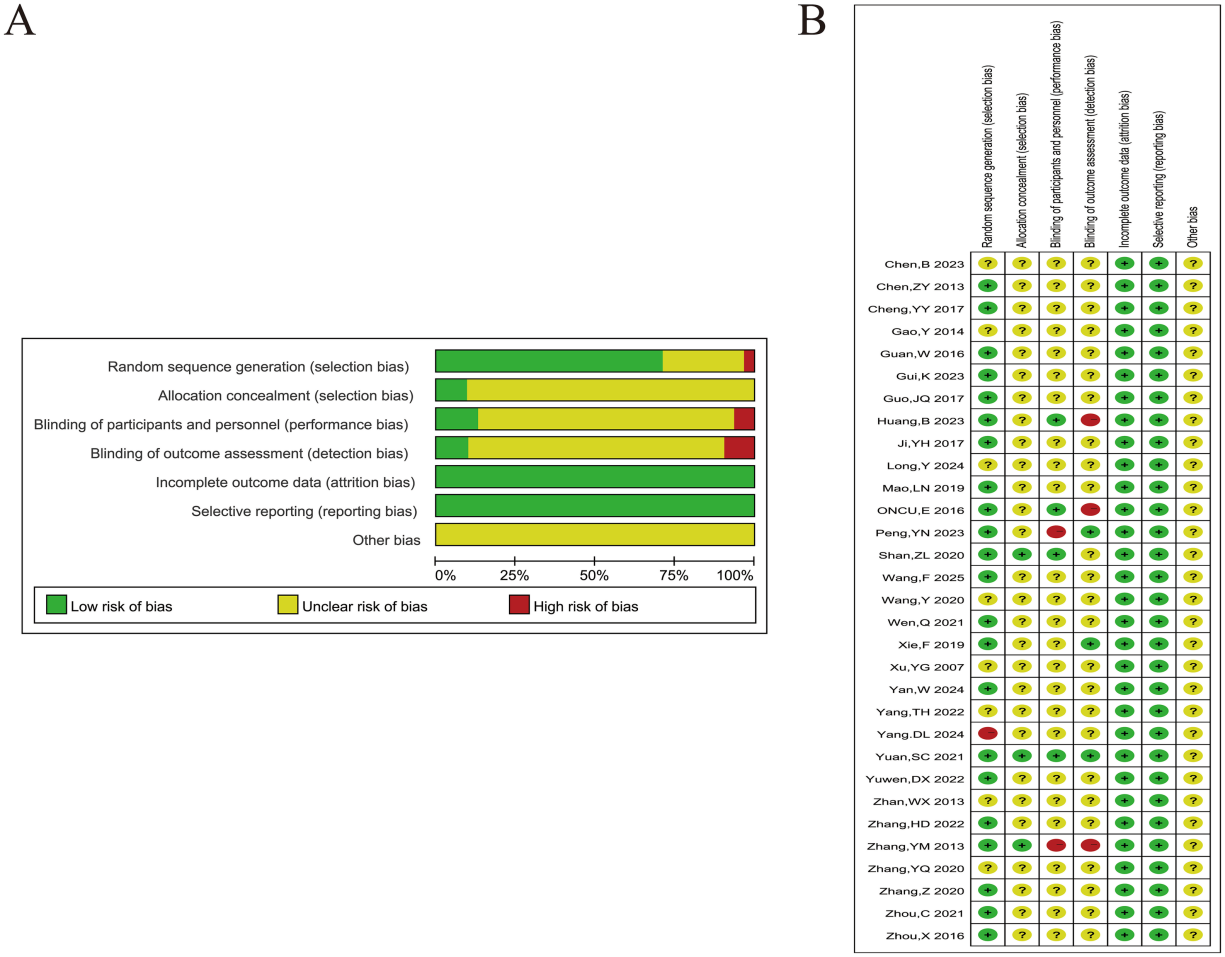
Risk of bias analysis. **(A)** Risk of bias summary; **(B)** Literature risk assessment diagram.

7 studies were deemed “unclear” due to the absence of specific methods and only the use of “random” words. 1 study was grouped according to treatment regimen and rated as “high risk” in the randomized sequence generation. 3 studies have clarified the allocation of hidden methods, which were considered “low risk.” However, only 6 studies have provided an interpretation of the blind method due to the unique nature of acupuncture as an auxiliary treatment (Figure 2).

### 3.4 Quality of evidence

We evaluated the quality of evidence using the GRADE classification. The quality of evidence for most outcomes was downgraded to low due to a high risk of bias and inconsistency across studies. Specifically, some studies lacked proper randomization, blinding, and allocation concealment, which contributed to the risk of bias. Additionally, inconsistency in the treatment protocols and participant characteristics across studies further impacted the reliability of the findings (Supplementary material 4). The overall certainty of the assessment is rated as moderate, low, or very low, depending on the quality of the individual studies and the presence of methodological limitations such as small sample sizes and unclear reporting of key methodological details.

### 3.5 Outcomes

#### 3.5.1 Effective rate

**Figure 3:**
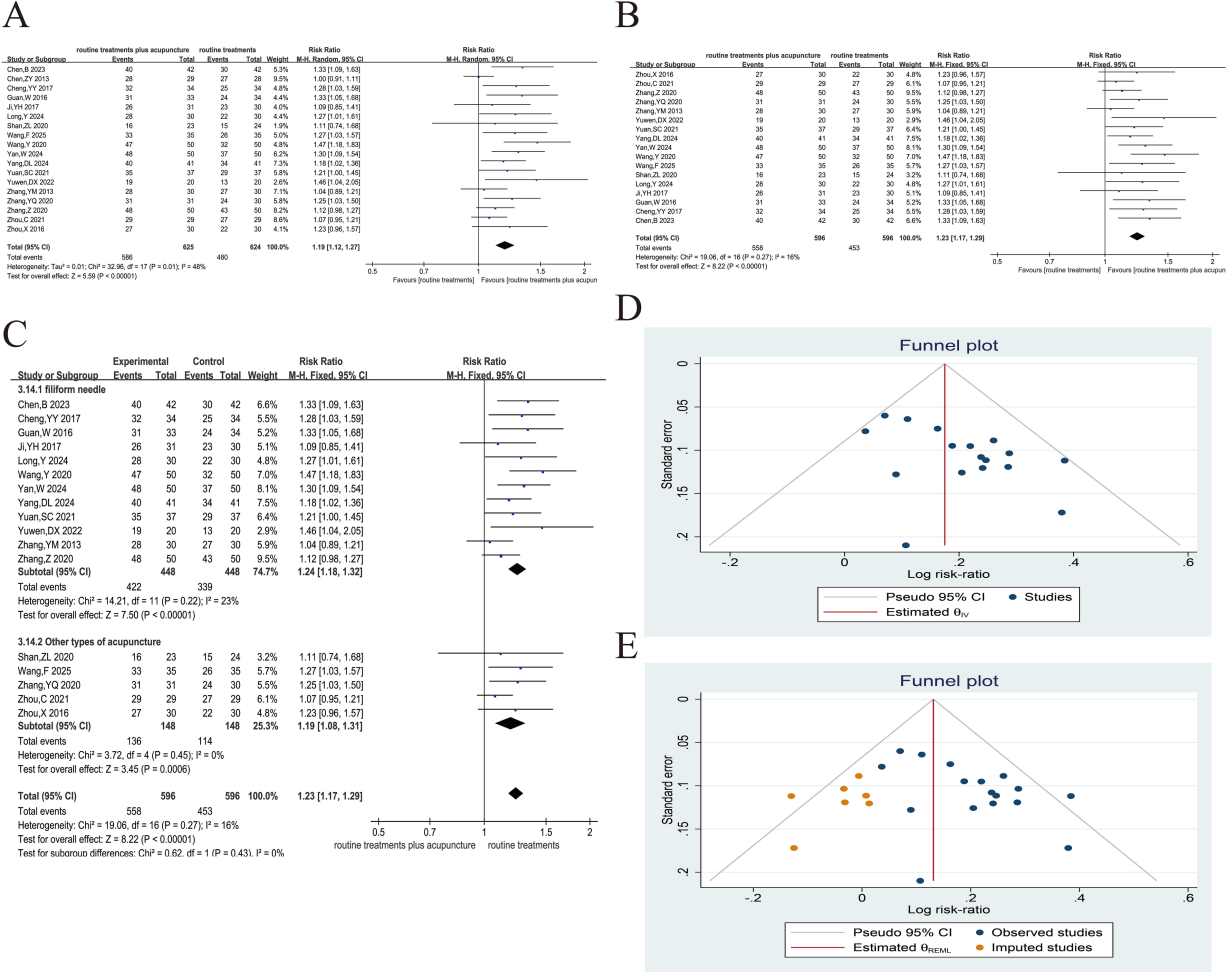
Acupuncture plus routine treatment versus routine treatment with effective rate. **(A)**: Forest plot of effective rate; **(B)**: Forest plot of effective rate after removing Chen’s study; **(C)**: Subgroup analysis of acupuncture modalities; **(D)**: Funnel plot of effective rate; **(E)**: Funnel plot of effective rate after applying trim and fill method.

Among the 31 articles, 18 reported the effective rate of treatment (8, 31, 33–48). Due to the different literature referring to the criteria for the formulation of efficacy, the criteria for the formulation of “clinical control,” “obvious effect” and “effective” were also slightly different, but the definitions of “ineffective” tended to be consistent (ineffective: The clinical symptoms, signs, and lung function of the patients were not substantially improved or aggravated, and the symptom score was reduced by < 30%). The effective rate = (overall cases − invalid cases)/overall cases × 100%. The heterogeneity test (Figure 3A) yielded results of (*p* = 0.01 and *I*^2^ = 48%), which suggested variability in results. The sensitivity analysis showed that after excluding the studies of Chen et al. (39), (*p* = 0.27, *I*^2^ = 16%), it indicated the elimination of heterogeneity. Using TCM pulse changes to select acupuncture meridians and acupoints may explain the analysis. The selection of acupuncture points varies from individual to individual; other articles normally used a limited selection of predetermined acupuncture points for treatment based on symptoms. The fixed effects model was used after excluding this study. As evident from the results, the experimental group’s therapeutic effect was considerably better in contrast to the control group, as evidenced by the substantial variation between both groups (RR = 1.23, 95%CI 1.17 ~ 1.29, *p* < 0.001) (Figure 3B). The purpose of the funnel plot was to determine whether there was any publication bias in this study (Figure 3D). When the funnel plot is tested for symmetry, the result of Egger’s test is *p* = 0.0139, so we carried out the trim and fill method (Figure 3E). The asymmetry of the funnel plot may slightly overestimate the possible effect of the experimental group. However, the asymmetry of the funnel plot has a negligible effect on publication bias and does not affect the conclusions of the meta-analysis. In all the studies, 1 used electroacupuncture, 1 used auricular acupuncture, 1 used intradermal acupuncture, 2 used Fu’s acupuncture, and the others were manual filiform needles. We also conducted a subgroup analysis according to the type of acupuncture to compare the overall efficacy of manual filiform acupuncture and other types of acupuncture, and the results showed that there was no significant difference in efficacy between them (*p* > 0.05) (Figure 3C).

#### 3.5.2 Lung function

##### 3.5.2.1 FEV_1_%

**Figure 4:**
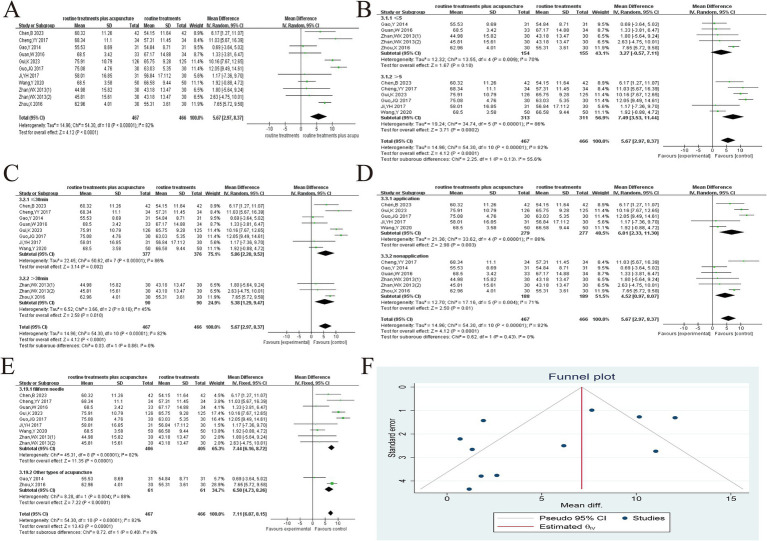
Acupuncture plus routine treatment versus routine treatment with FEV_1_%. **(A)**: Forest plot of FEV_1_%; **(B)**: Subgroup analysis of FEV_1_% (point stimulation amount); **(C)**: Subgroup analysis of FEV_1_% (point stimulation time); **(D)**: Subgroup analysis of FEV_1_% (acupuncture manipulation); **(E)**: Subgroup analysis of FEV_1_% (acupuncture modalities); **(F)**: Funnel plot of FEV_1_%.

The changes in FEV_1_% before and after treatment were documented in 11 studies across 10 articles (22, 24, 33–36, 42, 44, 49, 50). The heterogeneity test results were (*p* < 0.1, *I*^2^ > 50%). We conducted sensitivity analysis by individually eliminating each study. No study was identified as having a substantial influence on heterogeneity; therefore, the random effects model was implemented for the combined analysis. There was a significant variance between both groups (MD = 5.67, 95% CI 2.97 ~ 8.37, *p* < 0.001), suggesting that the experimental group can enhance the FEV_1_% of AECOPD patients in contrast to the control group (Figure 4A). Subgroup analysis was performed according to the amount of acupoint stimulation (Figure 4B), duration of needle retention or stimulation (Figure 4C), whether acupuncture manipulation was used (Figure 4D), and acupuncture modalities (Figure 4E). The results suggested that heterogeneity may be related to duration of needle retention or stimulation. In addition, the subgroup analysis also suggested that increasing the number of acupoint stimulations and the application of acupuncture manipulations could improve the efficacy. This study was examined for publication bias *via* the funnel plot (Figure 4F). Symmetry of the funnel plot was assessed using Egger’s test (*p* = 0.066), indicating no evidence of publication bias in the current analysis.

##### 3.5.2.2 FEV_1_/FVC

**Figure 5:**
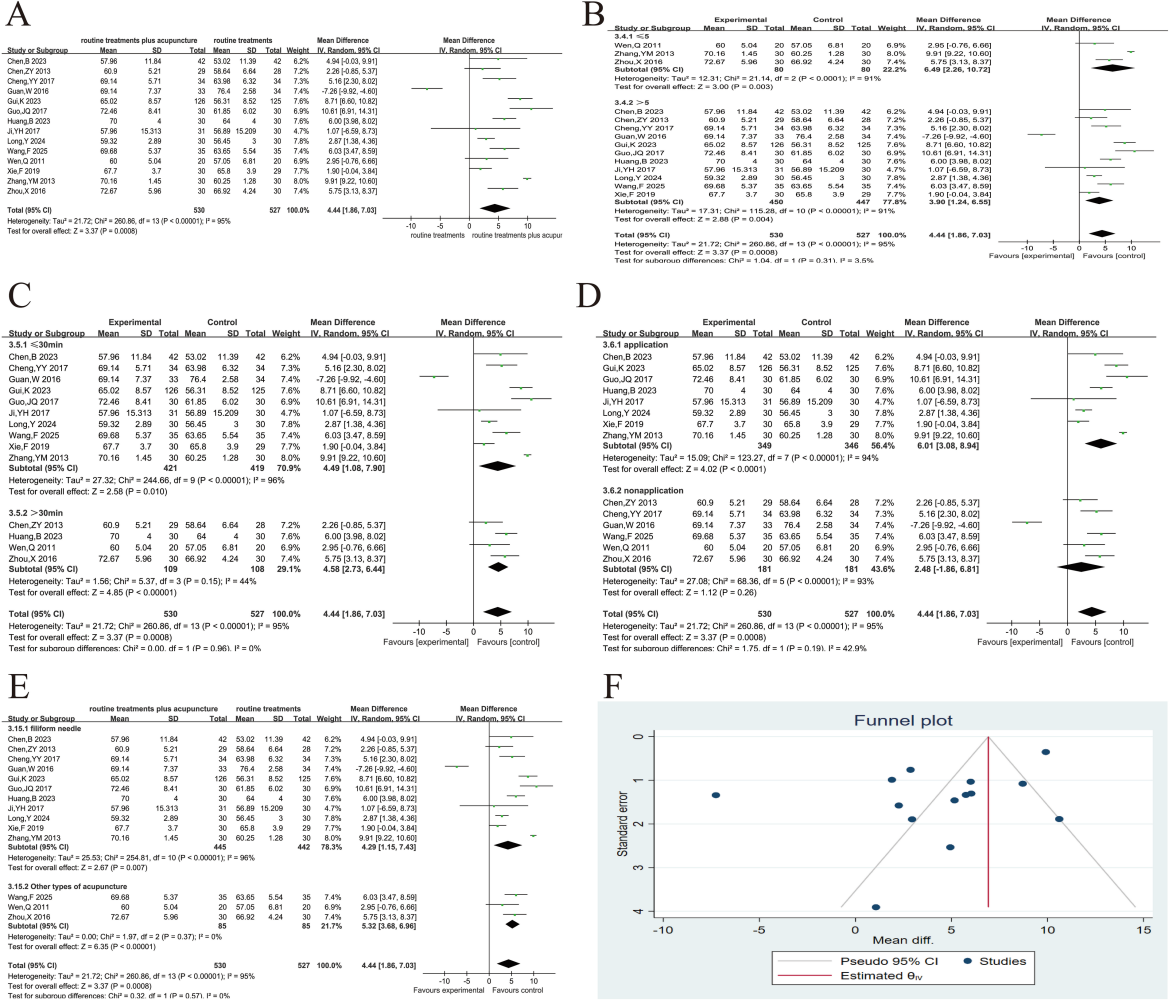
Acupuncture plus routine treatment versus routine treatment with FEV_1_/FVC. **(A)**: Forest plot of FEV_1_/FVC; **(B)**: Subgroup analysis of FEV_1_/FVC (point stimulation amount); **(C)**: Subgroup analysis of FEV_1_/FVC (point stimulation time); **(D)**: Subgroup analysis of FEV_1_/FVC (acupuncture manipulations); **(E)**: Subgroup analysis of FEV_1_/FVC (acupuncture modalities); **(F)**: Funnel plot of FEV_1_/FVC.

14 articles (22, 32–34, 36, 39, 43, 44, 47–52) reported before and after treatment changes in FEV_1_/FVC and heterogeneous results. There was considerable heterogeneity among these studies (*p* < 0.1, *I*^2^ > 50%) (Figure 5A). The random effects model was employed for the combined analysis, as no studies with a significant impact on heterogeneity were identified during the sensitivity analysis, which was conducted by systematically excluding each study. The experimental group was able to substantially enhance the FEV_1_/FVC of AECOPD patients in contrast to the control group, as evidenced by the significant variance between both groups (MD = 4.44, 95% CI 1.43 ~ 7.44, *p* < 0.01), Subgroup analysis suggested that both stimulation time and acupuncture modalities may be the reasons for heterogeneity, and also suggested that increasing the number of stimulation points and the application of acupuncture manipulations could improve the efficacy (Figures 5B–E). This study was examined for publication bias using the funnel plot. The funnel plot symmetry test was conducted, and Egger’s Test (*p* > 0.05) suggests that the study under examination in this article fails to demonstrate any publication bias (Figure 5F).

##### 3.5.2.3 FEV_1_

**Figure 6:**
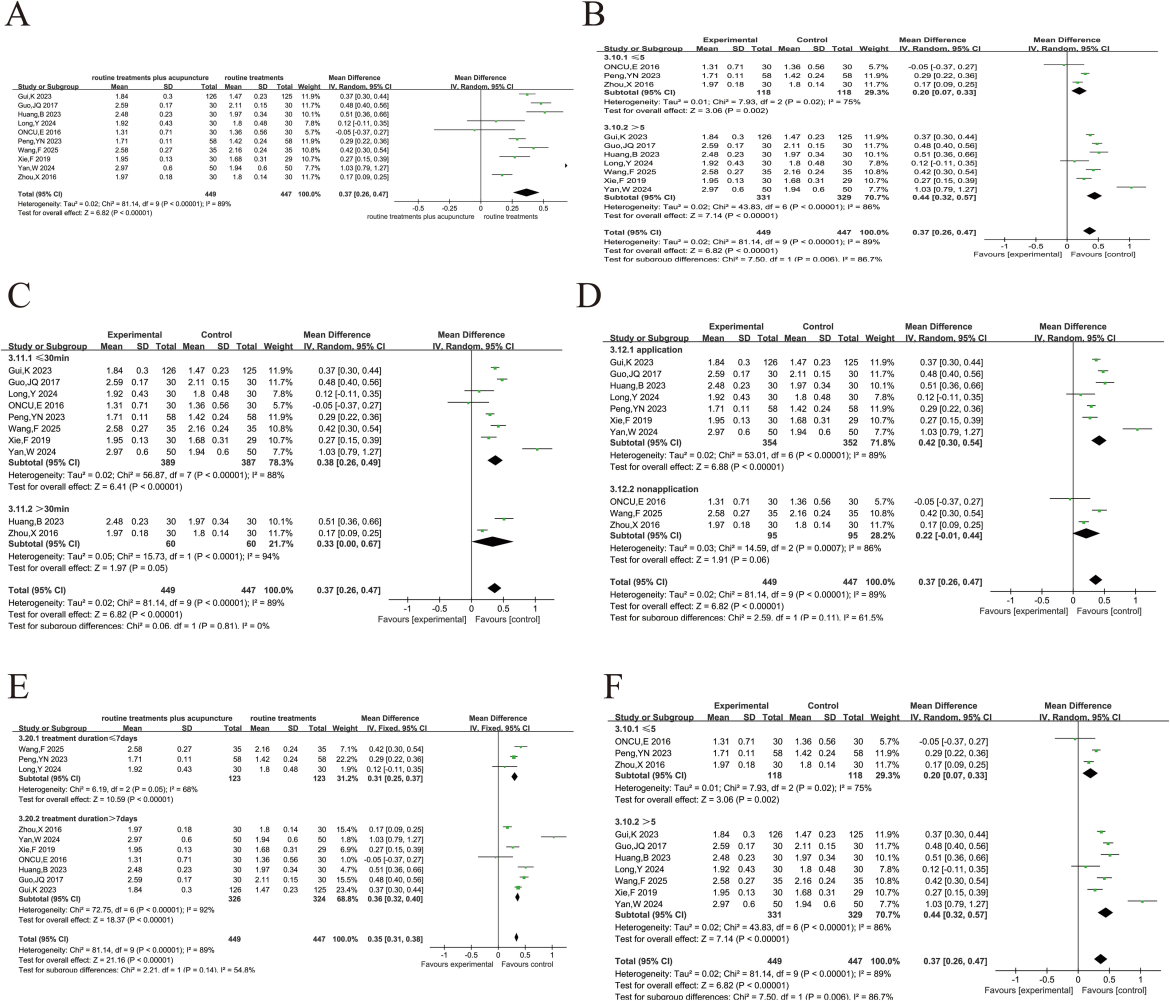
Acupuncture plus routine treatment versus routine treatment with FEV_1_. **(A)**: Forest plot of FEV_1_; **(B)**: Subgroup analysis of FEV_1_ (point stimulation amount); **(C)**: Subgroup analysis of FEV_1_ (stimulation time); **(D)**: Subgroup analysis of FEV_1_ (acupuncture manipulations); **(E)**: Subgroup analysis of FEV_1_ (treatment duration); **(F)**: Subgroup analysis of FEV_1_ (acupuncture modalities).

Based on the combined findings of 10 articles (22, 23, 36, 46–48, 50–53), patients receiving combined acupuncture and conventional western medical treatment show greater improvement compared to those receiving conventional western medical treatment alone (MD = 0.37, 95% CI 0.26 ~ 0.47, *p* < 0.001). Heterogeneity was found in 10 results (*p* < 0.1, *I*^2^ = 89%) (Figure 6A). Each article was removed one by one, and no significant heterogeneity was found, so a random effect model was used. Subgroup analysis of the studies did not reveal a reason for the heterogeneity, but the short duration of treatment may be a potential factor. As with the other two lung function outcome measures, increasing the number of acupoints and the application of acupuncture manipulations improved the efficacy (Figures 6B–F).

#### 3.5.3 Blood gas analysis

##### 3.5.3.1 PaO_2_

**Figure 7:**
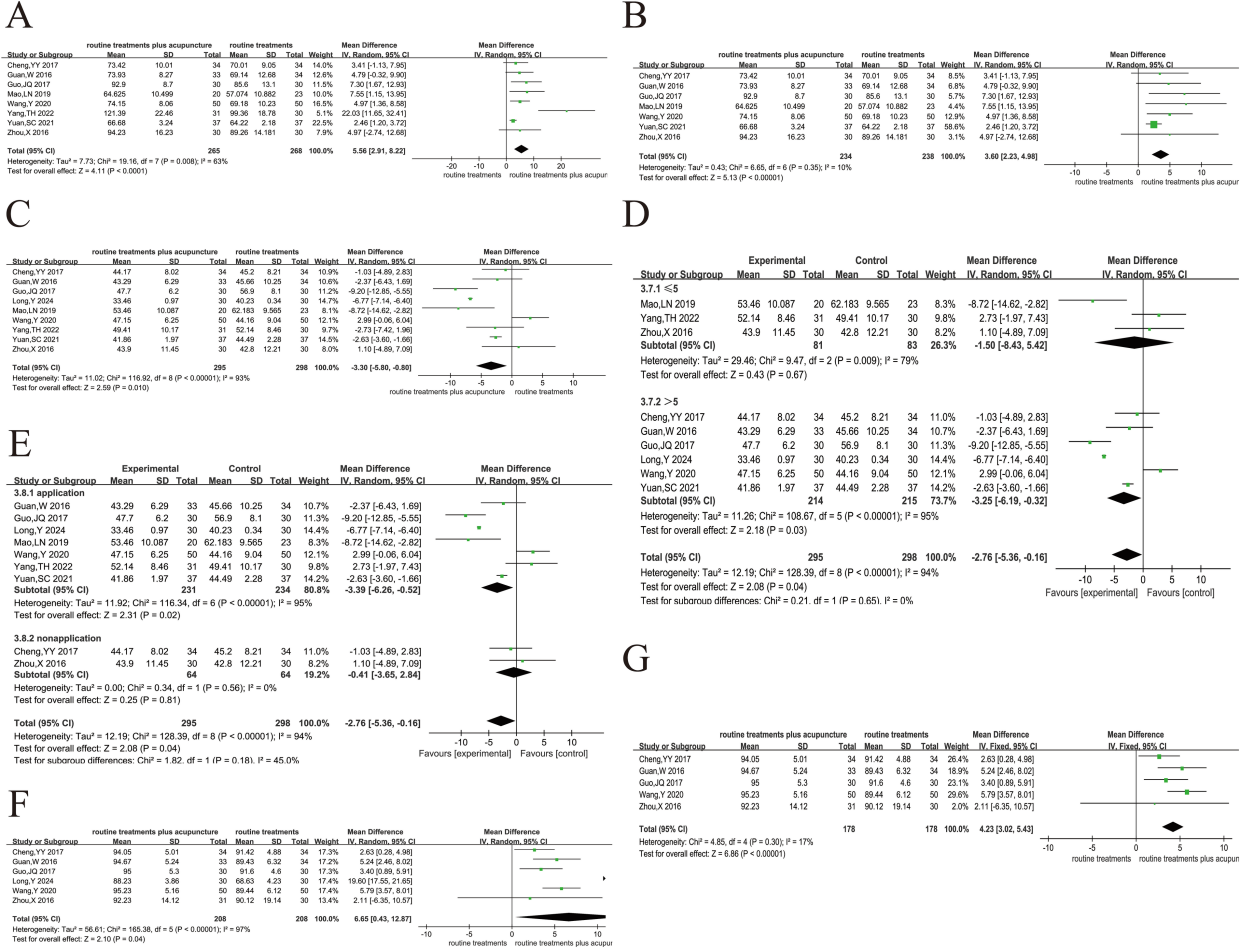
Acupuncture plus routine treatment versus routine treatment with blood gas analysis. **(A)**: Forest plot of PaO_2_; **(B)**: Forest plot of PaO_2_ after removing Yang’s study; **(C)**: Forest plot of PaCO_2_; **(D)**: Subgroup analysis of PaO_2_ (point stimulation amount); **(E)**: Subgroup analysis of PaO_2_ (acupuncture manipulation); **(F)**: Forest plot of SaCO_2_; **(G)**: Forest plot of SaCO_2_ after removing Long’s study_._

PaO_2_ was reported in 8 papers (8, 21, 33–36, 50, 54, 55) and the heterogeneity analysis (Figure 7A) indicated moderate heterogeneity (*p* < 0.008, *I*^2^ = 63%). Sensitivity analysis showed that excluding the study of Yang et al. (21), heterogeneity was eliminated (*p* = 0.35. *I*^2^ = 10%) (Figure 7B). Because invasive mechanical ventilation was used in this study, PaO_2_ could be better improved, while other studies used oxygen inhalation or non-invasive mechanical ventilation. The reason for this could be that Yang et al. used invasive mechanical ventilation, which is known to be more effective at improving PaO_2_ than oxygen inhalation or non-invasive mechanical ventilation used in the other studies. This difference in ventilation methods likely contributed to the heterogeneity. Additionally, Yang et al. did not provide a detailed explanation of their randomization method, while other studies used more clearly defined randomization techniques (e.g., random number tables). These methodological differences might have also contributed to the observed heterogeneity. Furthermore, Yang et al. included older patients (treatment group: 76.71 ± 7.84 years, control group: 77.77 ± 5.74 years), which could have influenced PaO_2_ response, adding to the heterogeneity. After the exclusion of Yang et al.’s study, a fixed-effects model was applied, which revealed a more significant improvement in PaO_2_ in the treatment group compared to the control group (MD = 3.60, 95% CI 2.23 ~ 4.98, *p* < 0.001). Although the exclusion of one study helped reduce heterogeneity, it raises concerns about the robustness of the overall conclusions. Future studies will adopt standardized ventilation strategies, clearer randomization methods and detailed baseline characteristic reports to better explain this variability.

##### 3.5.3.2 PaCO_2_

There were 9 articles (8, 21, 33–36, 48, 50, 54, 55) that discussed the results of PaCO_2_ (Figure 7C). The analyses showed a high degree of heterogeneity (*p* < 0.0001, *I*^2^ = 93%). Sensitivity analysis, by excluding each study individually, did not reduce heterogeneity significantly, so a random-effects model was used. The high heterogeneity observed suggests that variability in treatment methods, patient characteristics, and study design could be significant sources of the differences across studies. In all the studies, only Zhou et al. (36) was >30-min used needle retention or stimulation time >30-min, while the other studies used ≤30-min. Additionally, Zhou et al. (36) used Fu’s acupuncture treatment; and Oncu et al. (23) used transcutaneous electrical stimulation treatment, both of which differed from standard acupuncture techniques. These differences in acupuncture manipulation and acupuncture type were identified as potential sources of heterogeneity in the subgroup analysis (Figure 7E). Further subgroup analysis based on the number of acupoints did not reduce the heterogeneity (Figure 7D). The treatment group showed a better reduction in PaCO_2_ compared to the control group (MD = −3.30, 95% CI -5.80 ~ −0.80, *p* < 0.05). Future research should focus on standardizing treatment protocols, improving reporting of study methodologies, and considering key patient characteristics such as age and baseline health status. These steps can help reduce variability and improve the consistency of results across studies, allowing for more accurate comparisons and clearer conclusions.

##### 3.5.3.3 SaCO_2_

In 6 studies (33–36, 48, 50), approximately 416 cases were reported for SaO_2_ levels (Figure 7F). The results showed a high degree of heterogeneity among the 6 studies (*p* < 0.01, *I*^2^ = 97%). The heterogeneity was eliminated after the study by Long et al. (48), as the treatment duration was only 7 days in that study and at least 2 weeks in the other studies (Figure 7G). The addition of acupuncture had a substantial effect on the improvement of SaO_2_ in AECOPD patients (MD = 4.23, 95% CI 3.02 ~ 5.43, *p* < 0.001).

#### 3.5.4 Sports endurance and quality of life

##### 3.5.4.1 6MWT

**Figure 8:**
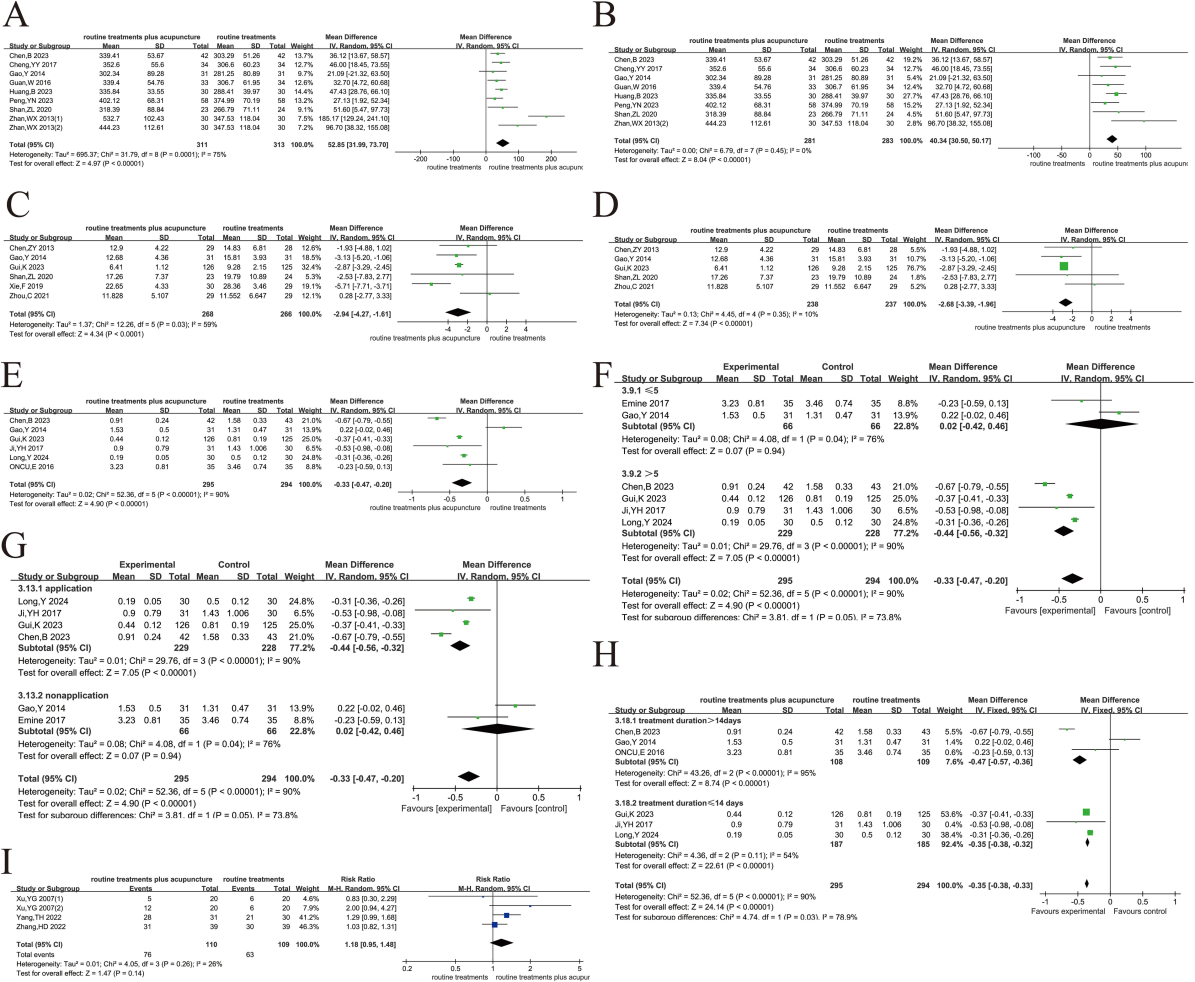
Acupuncture plus routine treatment versus routine treatment with sports endurance and quality of life. **(A)**: Forest plot of 6MWT; **(B)**: Forest plot of 6MWT after removing Zhan’s study (1); **(C)**: Forest plot of CAT; **(D)**: Forest plot of CAT after removing Xie’s study; **(E)**: Forest plot of mMRC; **(F)**: Subgroup analysis of mMRC (point stimulation amount); **(G)**: Subgroup analysis of mMRC (acupuncture manipulation); **(H)**: Subgroup analysis of mMRC (treatment duration); **(I)**: Forest plot of success rate of weaning.

This index includes 9 trials (24, 33, 34, 40, 44, 49, 52, 53), and the analysis indicated that there was large heterogeneity in the 9 studies (*p* < 0.0001, *I*^2^ = 75) (Figure 8A). The high heterogeneity was likely due to differences in study designs, treatment methods, and sample populations, which may have contributed to variability in the results. There was a substantial reduction in heterogeneity (*p* = 0.45, *I*^2^ = 0%) after the exclusion of Zhan et al.’s study (1, 49). We excluded Zhan et al.’s study due to significant methodological differences, including the use of coarse needle stimulation with stronger stimulation intensity, longer needle retention time (3 h), and the use of fewer acupoints compared to other studies that employed traditional filiform needles or press needles. These intervention differences likely accounted for much of the heterogeneity observed in the initial analysis. Additionally, Zhan et al. used invasive mechanical ventilation, which might have further contributed to the variability in outcomes compared to studies using oxygen inhalation or non-invasive mechanical ventilation.

We fully acknowledge that such baseline differences can affect exercise capacity and may introduce bias. Unfortunately, due to incomplete reporting of baseline characteristics in many of the included studies, we were unable to conduct a formal subgroup analysis based on disease severity or comorbidity status. Nevertheless, we carefully reviewed the available information and identified some common baseline features across studies. Specifically, most participants were male, which aligns with the current epidemiological trends of COPD. The average age was above 55 years, and the disease duration was approximately 10 years in several studies. These factors-older age, longer disease history, and male predominance-may have influenced baseline 6MWT performance and potentially contributed to inter-study variability. The findings depicted that acupuncture had a substantial impact on patients’ exercise endurance (MD = 40.34, 95% CI 30.50 ~ 50.17, *p* < 0.001) (Figure 8B). After excluding Zhan et al.’s study, the results showed a more consistent effect across the remaining studies, providing stronger evidence for the beneficial impact of acupuncture on exercise endurance.

In response to the heterogeneity observed, we attempted to explore potential sources by conducting subgroup analyses based on treatment duration, stimulation intensity, and manual acupuncture techniques. These factors were selected to reflect the clinical characteristics of acupuncture practice and the treatment features reported in the included studies. However, we fully acknowledge that these variables were not consistently or clearly defined across the studies, which limits the explanatory power of our subgroup analyses. Therefore, we present these analyses as hypothesis-generating rather than confirmatory, with the aim of offering potential insights for future research and trial design. Future trials need to provide detailed and standardized reports on the characteristics of participants and clearly define treatment parameters such as needle type, stimulation intensity, and treatment duration to facilitate subgroup analyses. This will help reduce heterogeneity and allow for a more accurate interpretation of the results.

##### 3.5.4.2 CAT

The meta-analysis included 6 trials (22, 24, 39–41, 51) that evaluated CAT. The experiment exhibited heterogeneity across different studies (*p* < 0.03, *I*^2^ = 59%) (Figure 8C). After the exclusion of Xie et al.’s studies (51), the heterogeneity analysis revealed contradictory results (*p* < 0.35, *I* ^2^ = 10%) (Figure 8D). Heterogeneity was considered due to the use of warm acupuncture therapy in this study and traditional acupuncture therapy in other studies. Warm acupuncture has the function of warming channels and collaterals and helping healthy qi. After deleting this study, a fixed effects model was used to merge. The findings illustrated a substantial reduction in the CAT score for the group receiving assisted acupuncture treatment in contrast to the group receiving conventional treatment. Further, patients experienced improvements in their QoL (MD = −2.68, 95% CI -3.39 ~ −1.96, *p* < 0.05).

##### 3.5.4.3 mMRC

The analysis of 6 articles (22–24, 42, 44, 48) found a high degree of heterogeneity (*p* < 0.001, *I*^2^ = 90%), which was not significantly reduced when each study was excluded one by one (Figure 8E). We attempted to explore potential sources of heterogeneity by conducting subgroup analyses based on treatment duration, stimulation intensity, and manual acupuncture techniques. These factors were selected to reflect the clinical characteristics of acupuncture practice and the treatment features reported in the included studies. However, these variables were not consistently or clearly defined across the studies, which limits the explanatory power of our subgroup analyses. Consequently, we present these analyses as hypothesis-generating rather than confirmatory, with the goal of providing insights for future research and trial design. Of all 6 studies, only the study of Oncu, E. (23) used transcutaneous electrical stimulation with needle retention or stimulation for > 30-min, and no significant reduction in heterogeneity was observed after removal. Therefore, subgroup analysis according to the amount of acupoint stimulation (Figure 8F), whether acupuncture manipulation was applied (Figure 8G), and treatment duration (Figure 8H) showed that treatment duration may be a source of heterogeneity.

The pooled effect size (MD = −0.33, 95%CI -0.47 ~ 0.20, *p* < 0.001) suggests a statistically significant benefit of acupuncture on breathlessness. However, this improvement is close to or below the minimal clinically important difference (MCID) for the mMRC scale, which suggests that the clinical significance may be limited. We believe that the limited improvement in mMRC scores may be partly attributable to the older age and longer disease duration of the participants in the included studies—factors known to affect exercise tolerance and dyspnea perception. Unfortunately, due to incomplete reporting, we were unable to further stratify the analysis by age or disease severity. Future trials should report detailed baseline characteristics and adopt standardized intervention protocols to enhance the consistency and interpretability of the results. These steps will help reduce the potential sources of heterogeneity and provide more reliable evidence for the clinical efficacy of acupuncture.

#### 3.5.5 Success rate of weaning

The 14-day success rate of weaning off ventilators was discussed in 4 studies (21, 55, 56). There was no heterogeneity in the four studies (*p* < 0.26, *I*^2^ = 26%). The fixed-effect model was employed to conduct a meta-analysis, which revealed that the 14-day weaning success rate did not differ dramatically between the conventional treatment and its combination with acupuncture treatment (RR = 1.18, 95% CI 0.95 ~ 1.48, *p* = 0.14) (Figure 8I). The combined relative risk was not statistically significant but still shows a trend suggesting that acupuncture may offer some benefit. We acknowledge that the small sample sizes in the studies included in this analysis likely led to low statistical power, which could have resulted in missing moderate effects. This limitation may have reduced the ability to detect a more substantial impact of acupuncture. We are unable to clarify the consistency of these studies in terms of offline assessment, intervention timing and treatment plans. This lack of consistency may lead to changes in the results and affect the interpretation of the research findings. Future research should aim to increase sample sizes, standardize evaluation methods, and provide clearer reporting of study quality to improve the reliability of the findings.

### 3.6 Safety and adverse reaction reports

Approximately 14 studies evaluated safety measurements and identified adverse reactions. Among them, Huang et al. (52) documented three cases of adverse reactions. These reactions included one case of needle fainting, one case of difficulties falling asleep, and one case of stomach dilation.

## 4 Discussion

We conducted this systematic review and meta-analysis to more comprehensively evaluate the efficacy and safety of acupuncture in treating acute exacerbation of chronic obstructive pulmonary disease. A total of 31 studies were included in this study, involving 2,299 participants. The therapeutic goals of AECOPD are to improve acute exacerbations and complications. Studies have indicated that compared with conventional western medicine treatment alone, acupuncture adjuvant therapy can significantly improve the effective rate of clinical treatment of patients (*p* < 0.001). Among the 31 studies, electroacupuncture was used in 3 studies, Fu’s acupuncture in 2 studies, ear acupuncture in 1 study, intradermal acupuncture in 1 study, and manual filiform needle in the remaining 24 studies. We conducted subgroup analysis to compare the overall efficacy of manual filiform needle and other acupuncture types, and the results showed that there was no significant difference in the effective rate (*p* > 0.05). Lung function is an important indicator for the diagnosis and evaluation of COPD, which can objectively assess the degree of acute exacerbation of patients and predict the mortality of patients (57). In this study, we conducted a comprehensive analysis of the three indicators of FEV_1_%, FEV_1_/FVC, and FEV_1_, among which there was a large heterogeneity in the results of the study of FEV_1_. After the deletion of literatures one by one and the subgroup analysis, we failed to locate the source of heterogeneity. Considering the particularity of acupuncture treatment, the heterogeneity may be caused by clinical differences. Subgroup analysis of the three outcome indicators also showed that increasing the number of acupoints and the application of acupuncture manipulations could better improve the lung function of patients. Acute respiratory failure is the most common complication of AECOPD. Rapid improvement of blood oxygen partial pressure and reduction of carbon dioxide retention are key to treatment (58). The addition of acupuncture can significantly increase PaO_2_ and decrease PaCO_2_ (*p* < 0.05), and also has statistical significance in increasing SaO_2_. The heterogeneity of PaCO_2_ results was high, subgroup studies suggest that acupuncture category and non-application of acupuncture manipulation may be sources of heterogeneity. The dosage of 6MWT, CAT, and mMRC was used to evaluate the exercise endurance and quality of life of COPD patients. This study showed that all three outcomes were improved after acupuncture treatment. Study (59) showed that 6MWT was often used to assess the severity of lung function and exercise ability of AECOPD patients. With the aggravation of ventilation dysfunction and the progression of the disease in COPD patients, there will be changes in the glucose metabolic pathway in the body, resulting in lactic acid accumulation and skeletal muscle dysfunction, which will lead to the decline of motor function and the aggravation of dyspnea in patients. The results of this study showed that the experimental group was significantly different from the control group, but the heterogeneity of the results was high (*I*^2^ = 75%). After reading the original article for analysis, in a study by Zhan et al. (49), thick needles were used for treatment, which was different from traditional acupuncture in other studies. Heterogeneity was eliminated (*I*^2^ = 0%). The heterogeneity of CAT was mainly from the application of warm acupuncture, and the heterogeneity of mMRC results was also due to the short duration of acupuncture treatment. The study on offline success rate was not statistically significant between the two groups. In this study, we found that the application of acupuncture manipulation could improve their degree of improvement. It was speculated that the reason may be that the stress response appeared in the human body after the application of acupuncture manipulation, and the resulting excitement was transmitted to the sympathetic nerve center, thereby alleviating airway spasm and respiratory muscle fatigue, and better improving dyspnea and oxygen deficiency (60).

Previous studies have mainly focused on people with stable COPD (61, 62), or the evaluation of AECOPD has included less literature unable to demonstrate the advantages of acupuncture in treating AECOPD (25). In contrast, our study focused specifically on the acute exacerbation period, a critical time for treatment, which showed that the rate of lung function decline in patients increased by more than 95% after one acute moderate–severe exacerbation (3). In this study, we evaluated lung function and arterial blood gas analysis more comprehensively and added FEV_1_ and SaO_2_ outcome indicators. In addition, we also evaluated the offline success rate, which is a key indicator for patients to successfully transition from mechanical ventilation support to autonomous breathing and can directly reflect the recovery degree and clinical benefit of patients’ overall respiratory function. This study confirms the positive effects of previous studies (25), on acupuncture in improving CAT, PaO_2_, FEV_1_/FVC and PaCO_2_. However, comparing previous meta-studies yielded different conclusions regarding FEV_1_%, CAT and 6MWT. This study showed that acupuncture-assisted therapy could significantly improve lung function (FEV_1_% and FEV_1_/FVC) and could significantly improve patients’ 6-min walking distance and mMRC score. In the study of Gao (24) and Zhan et al. (49), the results of FEV_1_% did not improve, considering that the two studies were caused by the stimulation of a single acupoint and the small amount of stimulation. Gao et al. (24) suggested that 6 MW did not change, possibly also because the study stimulated only a single acupuncture point. The study by Oncu et al. (23) did not find an improvement in mMRC, mainly because the study used noninvasive transcutaneous electrical stimulation, while other studies used invasive acupuncture.

In addition to the efficacy of acupuncture in the treatment of AECOPD, the potential mechanism of acupuncture in the treatment of AECOPD remains to be explored. Inflammatory response not only exacerbates airway obstruction but also affects alveola-capillary gas exchange, leading to decreased lung function and insufficient oxygenation (18, 63). Acupuncture can significantly reduce inflammatory biomarkers in COPD patients. Acupuncture also inhibited SOCS3/JAK1/STAT3 signaling (64), negatively regulated TLR-4/NF-κB signaling pathway (65), inhibited the release of pro-inflammatory cytokines in COPD rats (66), and alleviated airway inflammation. Thereby improving lung function; at the same time, acupuncture can improve diaphragm function, promote oxygen and relieve arterial carbon dioxide retention, relieve clinical hypoxia symptoms (8), inhibit cell apoptosis, reduce oxidative stress, and increase blood oxygen saturation (67). Acupuncture treatment can also reduce airway resistance, improve red blood cell immune function (68), regulate *β*-endorphin in patients (69), improve lung ventilation, effectively prevent BMI decline in COPD patients, increase peripheral skeletal muscle metabolism. This intervention not only alleviates dyspnea in patients with COPD but also enhances their exercise endurance and quality of life (70).

In the 31 studies included in this analysis, the top 3 acupoints in terms of frequency of use were Feishu (BL13), Zhongwan (RN17), and Danzhong (RN12). Feishu (BL13) is the back-shu point of the Lung Meridian. Research (71) has shown that Feishu (BL13) regulates the qi mechanism of the upper burner, promotes the dissemination of lung qi, and facilitates the circulation and dispersal of stasis. Acupuncture at Feishu (BL13) can treat various lung diseases such as cough, asthma, and pulmonary distension. Danzhong (RN12) is also an important acupoint for treating respiratory system diseases, with the function of regulating the qi mechanism of the upper burner, promoting the circulation of meridians, regulating qi, and dispersing stasis (72). Studies have indicated that acupuncture at Danzhong (RN12) has a beneficial therapeutic effect on some respiratory diseases. Clinically, using acupuncture at Danzhong (RN12) to treat chronic bronchitis and asthma has shown good results (73, 74). Additionally, research by Wang et al. (75) found that acupuncture at Danzhong (RN12) can reduce serum inflammatory factors in patients with AECOPD. Zhongwan (RN17) is the confluent point of the Stomach Meridian of Foot Yangming. Since the Hand Taiyin Lung Meridian originates from the middle burner, the relationship between the two is close. Strengthening the spleen and stomach, transforming qi and blood, leads to sufficient lung qi. Therefore, we can treat diseases with weakened lung functions that spread and descend by targeting Zhongwan (RN17) (76). Research (77) has shown that when Zhongwan (RN17) and Feishu (BL13) are combined, they can alleviate cough, transform phlegm, relieve asthma, and improve lung function in patients. The combined treatment of these three acupoints for AECOPD also clinically validates the traditional Chinese medicine theories of “the lung governs qi” and “nourishing the earth to produce metal.”

There are several study limitations of this review. (1) Heterogeneity of included studies: The included trials exhibited significant heterogeneity in terms of acupuncture treatment protocols, patient characteristics, and outcome measures. Such variability may affect the consistency of the results and limit the generalizability of our findings. In particular, differences in the acupuncture points used, needle insertion techniques, and treatment durations could contribute to the observed discrepancies. (2) Risk of bias: Many of the included studies presented unclear or high risk of bias, especially in terms of randomization and allocation concealment. Such practices could have introduced biases in the selection and reporting of outcomes. While we conducted a risk-of-bias assessment using the Cochrane tool, the limited quality of some studies could have affected the robustness of our conclusions. (3) Short treatment duration: The majority of the included studies had a treatment duration of only 1–2 weeks, which may not be sufficient to evaluate the long-term effects of acupuncture. Therefore, the potential benefits of acupuncture may have been underestimated in terms of sustained improvement or relapse prevention. (4) Limited follow-up and assessment of long-term effects: None of the included studies included long-term follow-up beyond the treatment period, which limits our understanding of the enduring effects of acupuncture on AECOPD patients. Future studies should incorporate long-term follow-up to assess whether acupuncture provides sustained benefits over time. Despite these limitations, our review indicates that acupuncture may be an effective adjunctive treatment for AECOPD. However, to strengthen the evidence base, future randomized controlled trials should focus on standardizing acupuncture protocols, extending treatment durations, and implementing long-term follow-up.

## 5 Conclusion

This systematic review and meta-analysis demonstrate that acupuncture, as an adjunctive treatment for acute exacerbations of chronic obstructive pulmonary disease, improves clinical efficacy and key outcomes. Our findings are consistent with previous studies that demonstrated improvements in the COPD Assessment Test (CAT) and arterial blood gas parameters (PaO2 and PaCO2). Unlike previous meta-analyses, the present study showed that adjunctive acupuncture significantly improved patient lung function FEV_1_% outcomes and significantly improved patient 6-min walk distance and modified Medical Research Council (mMRC) score; however, there was no significant difference in the success rate of weaning between the two groups. Although the review highlights clinical benefits, the heterogeneity of the included studies and the overall quality of the evidence suggest that more high-quality randomized controlled trials are needed to validate these findings and optimize treatment strategies. These studies should also prioritize standardizing acupuncture regimens, extending treatment duration, and conducting long-term follow-up assessments.

## 6 Future recommendations

Future research should aim to conduct larger, high-quality randomized controlled trials with consistent acupuncture protocols to evaluate its long-term effects on AECOPD outcomes. Additionally, exploring the underlying mechanisms of acupuncture’s therapeutic effects will enhance our understanding of its potential role in managing AECOPD. Comparative studies with conventional pharmacological treatments could further clarify the added value of acupuncture in clinical practice.

## Data Availability

The original contributions presented in the study are included in the article/Supplementary material, further inquiries can be directed to the corresponding author.

## References

[1] Global Initiative for Chronic Obstructive Lung Disease (GOLD). Global strategy for prevention, diagnosis and management of chronic obstructive pulmonary disease: 2025 report. (2024). Available online at: http://www.goldcopd.org/2025-gold-report/ (accessed November 11, 2024).

[2] Shah CH Onukwugha E Zafari Z Villalonga-Olives E Park JE Slejko JF. Economic burden of comorbidities among COPD patients hospitalized for acute exacerbations: an analysis of a commercially insured population. Expert Rev Pharmacoecon Outcomes Res. (2022) 22:683–90. doi: 10.1080/14737167.2021.1981291, PMID: 34530664

[3] Halpin DMG Decramer M Celli BR Mueller A Metzdorf N Tashkin DP. Effect of a single exacerbation on decline in lung function in Copd. Respir Med. (2017) 128:85–91. doi: 10.1016/j.rmed.2017.04.013, PMID: 28610675

[4] Nordon C Rhodes K Quint JK Vogelmeier CF Simons SO Hawkins NM . Exacerbations of Copd and their outcomes on cardiovascular diseases (Exacos-cv) Programme: protocol of multicountry observational cohort studies. BMJ Open. (2023) 13:e070022. doi: 10.1136/bmjopen-2022-070022, PMID: 37185641 PMC10151875

[5] Rothnie KJ Müllerová H Smeeth L Quint JK. Natural history of chronic obstructive pulmonary disease exacerbations in a general practice-based population with chronic obstructive pulmonary disease. Am J Respir Crit Care Med. (2018) 198:464–71. doi: 10.1164/rccm.201710-2029OC, PMID: 29474094 PMC6118021

[6] Expert group on management of acute exacerbation of chronic obstructive pulmonary disease. Expert consensus on the acute exacerbation of chronic obstructive pulmonary disease in China (revision in 2023). Int J Respir. (2023) 43:132–49. doi: 10.3760/cma.j.cn131368-20221123-01066

[7] Liu CG Li J. Effects of Yue Mei Jia Banxia decoction combined with salmeterol fluticasone in the treatment of acute exacerbation of chronic obstructive pulmonary disease. Practical integrated traditional Chinese and western medicine clinical practice. (2025) 25:49–51. doi: 10.13638/j.issn.1671-4040.2025.06.015 (in Chinese).

[8] Yuan S Huang X Hua S Zhou Y Rui Q. Using the ultrasonic detection evaluation of acupuncture for acute exacerbation of chronic obstructive pulmonary disease period II type diaphragmatic muscle function in patients with respiratory failure. Chin Acupunct Moxibust. (2021) 41:704–10. doi: 10.13703/j.0255-2930.20200606-k0007

[9] Hong H Huang C Chen C Zhou R Li J Liu J . Efficacy and safety of acupoint autohemotherapy in treating stable chronic obstructive pulmonary disease: protocol for a systematic review and meta-analysis. Medicine (Baltimore). (2019) 98:e17291. doi: 10.1097/MD.0000000000017291, PMID: 31568014 PMC6756693

[10] Wu JP Liu Y Bai L Xing AP. Application of Dachengqi decoction modified enema in acute exacerbation of chronic obstructive pulmonary disease. China Nat. (2019) 27:28–9. doi: 10.19621/j.cnki.11-3555/r.2019.0715

[11] Zhang P Zhang Y Guo M. Efficacy of Acupuncture in Treating Nape Back Myofascial Pain Syndrome: A Comprehensive Systematic Review and Meta-Analysis. J. Pain Res. (2025) 18:1667–81. doi: 10.2147/jpr.S509967, PMID: 40176784 PMC11963815

[12] Ang L Kim HJ Heo JW Choi TY Lee HW Kim JI . Acupuncture for the treatment of trigeminal neuralgia: a systematic review and Meta-analysis. Complement Ther Clin Pract. (2023) 52:101763. doi: 10.1016/j.ctcp.2023.101763 37159979

[13] Yao L Liu Y Li M Zheng H Sun M He M . The central regulatory effects of acupuncture in treating primary insomnia: a review. Front Neurol. (2024) 15:1406485. doi: 10.3389/fneur.2024.1406485, PMID: 39719980 PMC11666528

[14] Wang Y Du R Cui H Zhang L Yuan H Zheng S. Acupuncture for acute migraine attacks in adults: a systematic review and Meta-analysis. BMJ Evid Based Med. (2023) 28:228–40. doi: 10.1136/bmjebm-2022-112135, PMID: 37419658

[15] Liu YF Chen XG Liang FR Li Y Xiong C Qin EQ . Progress of Researches on Anti-Inflammatory Mechanism of Acupuncture Underlying Amelioration of Chronic Respiratory Diseases. J. Acupunct. Res. (2023) 48:147–52. doi: 10.13702/j.1000-0607.20220760 36858410

[16] Hu JS. Which 43 diseases are treated with acupuncture as announced and promoted by the WHO(World Health Organization)? J. Tradit. Chin. Med. Sci. (1989) 8: 57. doi: CNKI:SUN:ZZYZ.0.1989-08-034 (in Chinese).

[17] GOLD. Global strategy for the diagnosis, management, and prevention of chronic obstructive pulmonary disease: 2021 report; Global initiative for chronic obstructive lung disease. (2020) Available online at: https://goldcopd.org/wp-content/uploads/2020/11/GOLD-REPORT-2021-v1.1-25Nov20_WMV.pdf (Accessed November 17, 2020).

[18] Suzuki M Muro S Fukui M Ishizaki N Sato S Shiota T . Effects of acupuncture on nutritional state of patients with stable chronic obstructive pulmonary disease (COPD): re-analysis of COPD acupuncture trial, a randomized controlled trial. BMC Complement Altern Med. (2018) 18:287. doi: 10.1186/s12906-018-2341-3, PMID: 30355325 PMC6201549

[19] He YC Liu XM Guo JW. Effect of acupuncture on blood rheology of patients with chronic obstructive pulmonary disease of lung. Journal of Chinese Microcirculation. (2003) 7:387–388. doi: CNKI:SUN:ZWXH.0.2003-06-022

[20] Chen JF Luo WX Zhou HF. Effects of different acupuncture frequencies on respiratory function and immune status in patients with stable chronic obstructive pulmonary disease. World Chin Med. (2018) 13:2839–42. doi: 10.3969/j.issn.1673-7202.2018.11.043(in Chinese)

[21] Yang T Gao Z Wu Y. BoShi abdominal needle bleed air return element method in AECOPD application effect in the treatment of invasive mechanical ventilation withdraw machine. Res Integr Tradit Chin West Med. (2022) 14:230–3. doi: 10.3969/j.issn.1674-4616.2022.04.004

[22] Gui K. Exploring to the clinical value of acupuncture and moxibustion in the adjuvant treatment of chronic obstructive pulmonary disease based on NLRP3 and the balance of Th1 and Th2. Chin Foreign Med Res. (2023) 21:6–11. doi: 10.14033/j.cnki.cfmr.2023.25.002

[23] Oncu E Zincir H. The effect of transcutaneous electrical nerve stimulation in patients with acute exacerbation of chronic obstructive pulmonary disease: randomised controlled trial. J Clin Nurs. (2017) 26:1834–44. doi: 10.1111/jocn.13450, PMID: 27325551

[24] Gao Y Ma Y Sun L Li D Zhang Y Li S . Chronic obstructive pulmonary disease with acute aggravating period of acupuncture treatment of curative effect and evaluation research. Chin J Clin. (2014) 42:42–4. doi: 10.3969/j.issn.1674-4616.2022.04.004

[25] Yang C Tian H Xu G Luo Q Sun M Liang F. Efficacy of acupuncture in acute exacerbation of chronic obstructive pulmonary disease: a systematic review and Meta-analysis. Int J Chron Obstruct Pulmon Dis. (2024) 19:707–20. doi: 10.2147/COPD.S450257, PMID: 38495215 PMC10942019

[26] Lu C Wu LQ Hao H Kimberly Leow X Xu FW Li PP . Clinical efficacy and safety of acupuncture treatment of TIC disorder in children: a systematic review and meta-analysis of 22 randomized controlled trials. Complement Ther Med. (2021) 59:102734. doi: 10.1016/j.ctim.2021.102734, PMID: 33989798

[27] Giovanardi CM Gonzalez-Lorenzo M Poini A Marchi E Culcasi A Ursini F . Acupuncture as an alternative or in addition to conventional treatment for chronic non-specific low back pain: a systematic review and meta-analysis. Integr Med Res. (2023) 12:100972. doi: 10.1016/j.imr.2023.100972, PMID: 37637183 PMC10448023

[28] Higgins JPT Thomas J Chandler J. Cochrane hand-book for systematic reviews of interventions. Hoboken: Wiley-Blackwell (2008).

[29] Sterne JAC Savović J Page MJ Elbers RG Blencowe NS Boutron I . RoB 2: a revised tool for assessing risk of bias in randomised trials. BMJ. (2019) 366:l4898. doi: 10.1136/bmj.l4898 31462531

[30] Balshem H Helfand M Schünemann HJ Oxman AD Kunz R Brozek J . GRADE guidelines: 3. Rating the quality of evidence. J Clin Epidemiol. (2011) 64:401–6. doi: 10.1016/j.jclinepi.2010.07.015 21208779

[31] Yuwen DX (2022). Clinical observation and prognosis of Longsha Kaihe six-qi acupuncture in the adjuvant treatment of AECOPD patients with type II respiratory failure. [Master’s Thesis]. Tianjin: Tianjin University of Traditional Chinese.

[32] Wen Q Li L Yu P. Anti-asthma den percutaneous nerve electrical stimulation effect of lung function in patients with COPD acute phase: a randomized controlled study. Chin Acupunct Moxibust. (2011) 31:97–100. doi: 10.13703/j.0255-2930.2011.02.002 21442803

[33] Guan W Shang F Wang Y. Treatment of abdominal acupuncture in acute exacerbation of chronic obstructive pulmonary disease clinical randomized controlled study on respiratory muscle fatigue. J Emerg Tradit Chin Med. (2016) 25:594–7+615. doi: 10.3969/j.issn.1004-745X.2016.04.009

[34] Cheng YY. (2017) Clinical study of abdominal acupuncture in the treatment of respiratory muscle fatigue syndrome in acute exacerbation of chronic obstructive pulmonary disease. [Master’ Thesis]. Taiyuan: Shanxi University of Traditional Chinese Medicine.

[35] Wang Y Li C. Abdominal needle treatment of chronic obstructive pulmonary diseases acute exacerbation period (AECOPD) well fatigue analysis of the clinical efficacy of psychological issue. Psychol Mon. (2020) 15:218. doi: 10.19738/j.cnki.psy.2020.11.193

[36] Zhou X. (2016) Clinical study on the influence of pulmonary ventilation function. [Master’s Thesis]. Nanjing: Nanjing University of Traditional Chinese Medicine.

[37] Zhang YQ. Floating needle therapy in patients with chronic obstructive pulmonary disease exacerbations affect clinical research. Chin Community Doctors. (2020) 36:84–5. doi: 10.3969/j.issn.1007-614x.2020.02.049

[38] Zhao Z. Abdomen for chronic obstructive pulmonary diseases acute exacerbation stage well effect analysis of fatigue syndrome. Health Care Guide Health Guide. (2020) 4:42. Available at: https://d.wanfangdata.com.cn/periodical/Ch9QZXJpb2RpY2FsQ0hJTmV3UzIwMjUwMTE2MTYzNjE0EhF5c2Jqem4teDIwMjAwNDA0NRoIOG5wdTU3M24%3D

[39] Chen ZY. (2013) Pulse acupuncture treatment of chronic obstructive pulmonary disease (copd) patients with acute aggravating period clinical efficacy research. [Master’s Thesis]. Guangzhou: Traditional Chinese Medicine.

[40] Shan ZL. (2020) A comparative study on the clinical efficacy of two auricular acupuncture therapies in the treatment of acute exacerbation of chronic obstructive pulmonary disease. [Master’s Thesis]. Wuhan: Hubei University of Chinese Medicine.

[41] Zhou C. (2021) Clinical study of intradermal acupuncture regulating the liver and regulating the lung in the treatment of acute exacerbation of chronic obstructive pulmonary disease. [Master’s Thesis]. Beijing: Beijing University of Traditional Chinese Medicine.

[42] Ji YH. (2017) Clinical effect of Pingni Zhichuan principle needling in the treatment of acute exacerbation of chronic obstructive pulmonary disease. [Master’s Thesis]. Fuchou: Fujian University of Traditional Chinese Medicine.

[43] Zhang YM. (2013) Acupuncture or out side of chronic obstructive pulmonary disease in patients with acute aggravating period – phlegm dampness indicates pulmonary lung function, humoral immune clinical study. [Master’s Thesis]. Tianjin: Tianjin University of Chinese Medicine.

[44] Chen B Liu HY Sun LL Zhao ZX. Analysis of the improvement effect of acupuncture and moxibustion therapy on respiratory muscle fatigue in patients with acute exacerbation of chronic obstructive pulmonary disease. Chin J Mod Drug Appl. (2023) 17:152–5. doi: 10.14164/j.cnki.cn11-5581/r.2023.15.043

[45] Yang DL. Effect of Canggui-Tanxue acupuncture on acute exacerbation of chronic obstructive pulmonary disease and its influence on the level of inflammatory factors. Inner Mong J Tradit Chin Med. (2024) 43:118–20. doi: 10.16040/j.cnki.cn15-1101.2024.06.044

[46] Yan W Zhang PP. Clinical effect analysis of Bufei-Guben acupuncture as an assisted treatment of patients with acute exacerbation of chronic obstructive pulmonary disease. Mod Med Health Res Electron J. (2024) 8:106–8. doi: 10.3969/j.issn.2096-3718.2024.22.034

[47] Wang F Ma HH Yu JH Zhou LH. Efficacy observation of transcutaneous acupoint electrical stimulation combined with conventional drugs in the treatment of acute exacerbation of chronic obstructive pulmonary disease with phlegm turbidity obstructing lung syndrome and its effect on lung function and serum inflammatory factors. Chin J Tradit Med Sci Technol. (2025) 32:86–8. Available at: https://d.wanfangdata.com.cn/periodical/Ch9QZXJpb2RpY2FsQ0hJTmV3UzIwMjUwMTE2MTYzNjE0EhB6Z3p5eWtqMjAyNTAxMDIxGghheGM2b3drZg%3D%3D

[48] Long Y Liu YL Zhu SQ Wu BX Li F. Clinical effect of acupuncture combined with non-invasive ventilation in the treatment of acute exacerbation of chronic obstructive pulmonary disease with respiratory failure. Liaoning J Tradit Chin Med. (2024) 51:175–8. doi: 10.13192/j.issn.1000-1719.2024.04.045

[49] Zhan WX Wang JD Wu AP Wang XP. Effects of disease BODE index and quality of life on thick needle penetrating Shanzhong point on acute exacerbation of chronic obstructive pulmonary disease. J Basic Chin Med. (2013) 19:1187–9. doi: 10.19945/j.cnki.issn.1006-3250.2013.10.033

[50] Guo JQ Wang FD Chen JP. Clinical study on Pingchuan-Tiaozhong acupuncture in the treatment of AECOPD complicated with type IIrespiratory failure. Shandong J Tradit Chin Med. (2017) 36:773–6. doi: 10.16295/j.cnki.0257-358x.2017.09.012

[51] Xie F Wu YP Lei L Ren JG Zhang B. Warm acupuncture treatment of chronic obstructive pulmonary disease (copd) phlegm turbidity indicates pulmonary syndrome: a randomized controlled study. Chin Acupunct. (2019) 39:918–22. doi: 10.13703/j.0255-2930.2019.09.002 31544376

[52] Huang B Peng HX. Acupuncture catharsis treatment phlegm heat type indicates pulmonary chronic obstructive pulmonary disease exacerbations clinical observation. Mod Distance Educ Tradit Chin Med China. (2023) 21:117–9. doi: 10.3969/j.issn.1672-2779.2023.20.039

[53] Peng YN. (2023). Observation on the clinical efficacy of Shao’s five needle method in the treatment of acute exacerbation of chronic obstructive pulmonary disease. [Master’s Thesis]. Chengdu: Chengdu University of Traditional Chinese Medicine.

[54] Mao LN Chen J Xu Q Yang S. Comparative study on the early pulmonary rehabilitation effect of acupuncture and extracorporeal diaphragm pacing in AECOPD patients with type II respiratory failure. Guangming J Chin Med. (2019) 34:1231–3. doi: 10.3969/j.issn.1003-8914.2019.08.038

[55] Xu YG Lei S Xuan LH . Application value of Danzhong (CV 17) acupuncture needle in weaning from mechanical ventilation in patients with chronic obstructive pulmonary disease. Chin J Integr Tradit West Med Emerg Treat. (2007) 14:67–9. doi: 10.3321/j.issn:1008-9691.2007.02.001

[56] Zhang HD Jin KP Li XB. Effect of acupuncture on diaphragmatic thickening fraction in chronic obstructive pulmonary disease patients with mechanical ventilation. Chin Sci Technol J Database Med Health. (2022) 9:0144–8. Available at: https://qikan.cqvip.com/Qikan/Article/Detail?id=1000003543579&from=Qikan_Search_Index

[57] Rehman AU Shah S Abbas G Harun SN Shakeel S Hussain R . Assessment of risk factors responsible for rapid deterioration of lung function over a period of one year in patients with chronic obstructive pulmonary disease. Sci Rep. (2021) 11:13578. doi: 10.1038/s41598-021-92968-5, PMID: 34193949 PMC8245547

[58] The federation of world federation of Chinese medicine medical professional committee. Guidelines for the diagnosis and treatment of chronic obstructive pulmonary disease with integrated traditional Chinese and western medicine (2022 edition). Chin J Evid Based Med. (2023) 23:1117–28. doi: 10.7507/1672-2531.202304016

[59] Zhang MM Zhang Q Shu QQ Wang Y Zhu H. Effect evaluation of six-character breathing exercises assisted long-term home oxygen therapy in patients with chronic obstructive pulmonary disease. Chin J Pract Nurs. (2019) 35:2108–13. doi: 10.3760/cma.j.issn.1672-7088.2019.27.006

[60] Li YY Liu JP Shi SF Yang KZ Gong Y Sun J . Acupuncture with twirling reinforcing and reducing manipulation shows a control of hypertension and regulation of blood pressure-related target brain regions in spontaneously hypertensive rat: a preliminary resting-state functional Mri study. Front Neurosci. (2023) 17:1161578. doi: 10.3389/fnins.2023.1161578, PMID: 37304030 PMC10250630

[61] Ma YZ Zhang D Zhao GX Wang J Zhang HL. Acupuncture and Moxibustion in treatment of chronic obstructive pulmonary disease at stable stage: a network Meta-analysis. Chin Acupunct Moxibust. (2023) 43:843–53. doi: 10.13703/j.0255-2930.20220618-k0004 37429667

[62] Wei Y Yuan N Dong Y Wang L Ding J. Transcutaneous electrical nerve stimulation over Acupoint for chronic obstructive pulmonary disease: a systematic review and Meta-analysis. Front Public Health. (2022) 10:937835. doi: 10.3389/fpubh.2022.937835, PMID: 36276359 PMC9583392

[63] Stolz D Mkorombindo T Schumann DM Agusti A Ash SY Bafadhel M . Towards the elimination of chronic obstructive pulmonary disease: a lancet commission. Lancet. (2022) 400:921–72. doi: 10.1016/s0140-6736(22)01273-9, PMID: 36075255 PMC11260396

[64] Wu HH Pan Z Liu HY Zhang XF Xiang SY Xu SW . Electroacupuncture improves pulmonary function by reducing inflammatory reaction via inhibiting Mir-19b-3p to regulate Socs3/Jak1/Stat3 signaling pathway in mice with chronic obstructive pulmonary disease. Acupunct Res. (2024) 49:1248–56. doi: 10.13702/j.1000-0607.20231018, PMID: 39681482

[65] Wicherska-Pawłowska K Wróbel T Rybka J. Toll-Like Receptors (Tlrs), Nod-like receptors (Nlrs), and rig-I-like receptors (Rlrs) in innate immunity. Tlrs, Nlrs, and Rlrs ligands as immunotherapeutic agents for hematopoietic diseases. Int J Mol Sci. (2021) 22:13397. doi: 10.3390/ijms222413397, PMID: 34948194 PMC8704656

[66] Li Y Zhang XF Liu ZB Zhan WT Zhang Y Cheng C . Effect of Electroacupuncture on pulmonary function and M1 polarization of alveolar macrophages in rats with chronic obstructive pulmonary disease. Acupunct Res. (2020) 45:173–9. doi: 10.13702/j.1000-0607.190621 32202707

[67] Li D Wang L Shi S Deng X Zeng X Li Y . Ubiquitin-like 4a alleviates the progression of intracerebral hemorrhage by regulating oxidative stress and mitochondrial damage. Exp Anim. (2024) 73:421–32. doi: 10.1538/expanim.24-0035, PMID: 38852999 PMC11534490

[68] Peng XQ Li LF Liu J Liu ZQ Wu L Zhu XB. Effect of electroacupuncture at acupoints on airway resistance and oxygenation function in patients with chronic obstructive pulmonary disease during unilateral lung ventilation. New Chin Med. (2014) 46:180–2. doi: 10.13457/j.cnki.jncm.2014.07.084

[69] Ngai SP Jones AY Hui-Chan CW Ko FW Hui DS. An adjunct intervention for management of acute exacerbation of chronic obstructive pulmonary disease (AECOPD). J Altern Complement Med. (2013) 19:178–81. doi: 10.1089/acm.2011.0222, PMID: 22775329

[70] Ge Y Yao H Tong J He Y Li G Kong X. Effects of acupuncture on peripheral skeletal muscle exercise ability in patients with chronic obstructive pulmonary disease at stable phase. Chin Acupunct Moxibust. (2017) 37:366–71. doi: 10.13703/j.0255-2930.2017.04.005, PMID: 29231586

[71] Shen XY Liu CZ. Meridians learning. Beijing: Chinese Press of Traditional Chinese medicine (2021).

[72] Wang LL Zhang XZ. Clinical application of Danzhong point. Henan Tradit Chin Med. (2020) 40:1937–40. doi: 10.16367/j.issn.1003-5028.2020.12.0484

[73] Hou RR Xu SC Zhang XX Feng WJ Ou XH Wang XG. Therapeutic effect of acupoint sticking in summer and sequential plaster in winter on chronic bronchitis with spleen and kidney Yang deficiency. Shaanxi Tradit Chin Med. (2014) 35:394–6. doi: 10.3969/j.issn.1000-7369.2014.04.005

[74] Zhang GP Gao RR. Comparison of acupoint catgut embedding and aminophylline in the treatment of bronchial asthma. Shaanxi J Tradit Chin Med. (2017) 38:1392–3. doi: 10.3969/j.issn.1000-7369.2017.10.036

[75] Wang D Wu A Wang Y Diao S Zhan W. Effects of acupuncture on C-reactive protein during exacerbations of chronic obstructive pulmonary disease. Chinese Journal of Acupuncture and Moxibustion (Electronic Edition). (2015) 4:5–8.

[76] Li TP. (2014) Often point in qing dynasty and the qing dynasty before clinical application of law research. [Master’s Thesis]. Shangdong: Shandong University of Traditional Chinese Medicine.

[77] Gao YH Ma F Si DD. Moxibustion pulmonary shenshu point, chung wan acupuncture point in combination with small qinglong decoction clinical curative effect of chronic cough. Shenzhen Combine Tradit Chin West Med Mag. (2023) 33:34–7. doi: 10.16458/j.cnki.1007-0893.2023.13.011

