# Supplementary material for: Efficacy of acupuncture as adjunctive therapy for patients with acute exacerbation of chronic obstructive pulmonary disease: a systematic review and meta-analysis
Source: Front Med (Lausanne). 2025 May 12;12:1513888. doi: 10.3389/fmed.2025.1513888 (PMC12104076; doi:10.3389/fmed.2025.1513888)
Supplement: Supplementary file 2 [file Data_Sheet_2.docx]

| **Databases** | **Searches** | **Hits** |
| --- | --- | --- |
| **PubMed Database** | **#1**："pulmonary disease, chronic obstructive"[MeSH Terms]  **#2**："pulmonary disease chronic obstructive"[Title/Abstract] OR "chronic obstructive lung disease"[Title/Abstract] OR "chronic obstructive pulmonary diseases"[Title/Abstract] OR "COPD"[Title/Abstract] OR "COAD"[Title/Abstract] OR "chronic obstructive airway disease"[Title/Abstract] OR "chronic obstructive airway diseases"[Title/Abstract] OR "airflow obstruction chronic"[Title/Abstract] OR "airflow obstructions chronic"[Title/Abstract] OR "airflow obstruction chronics"[Title/Abstract] OR "chronic airflow obstructions"[Title/Abstract] OR "chronic airflow obstruction"[Title/Abstract] OR "lung diseases obstructive"[Title/Abstract]  **#3**：#1or #2  **#4**："Symptom Flare Up"[MeSH Terms] OR "Inpatients"[MeSH Terms]  **#5**："symptom flare up"[Title/Abstract] OR "recurrent disease"[Title/Abstract] OR "acute exacerbation"[Title/Abstract] OR "acute management"[Title/Abstract] OR "infective exacerbation"[Title/Abstract] OR "exacerbation"[Title/Abstract] OR "aggravation"[Title/Abstract] OR "inpatient"[Title/Abstract] OR "inpatients"[Title/Abstract] OR "hospital patient"[Title/Abstract] OR "hospital admission"[Title/Abstract] OR "hospitalization"[Title/Abstract] OR "rehospitalization"[Title/Abstract]  **#6**：#4 or #5  **#7**：#3 and #6  **#8**："acute exacerbation of chronic obstructive pulmonary disease"[Title/Abstract] OR "AECOPD"[Title/Abstract] OR "acute exacerbation of copd"[Title/Abstract] OR "exacerbation of copd"[Title/Abstract]  **#9**：#7 or #8  **#10**："Acupuncture"[MeSH Terms] OR "Acupuncture Therapy"[MeSH Terms]  **#11**："Acupuncture"[Title/Abstract] OR "acupuncture therapy"[Title/Abstract] OR "acupuncture points"[Title/Abstract] OR "body acupuncture"[Title/Abstract] OR "manual acupuncture"[Title/Abstract] OR "electroacupuncture"[Title/Abstract] OR "electro-acupuncture"[Title/Abstract] OR "warm acupuncture"[Title/Abstract] OR "Needle"[Title/Abstract] OR "Needling"[Title/Abstract] OR "Pricking"[Title/Abstract] OR "Bloodletting"[Title/Abstract] OR "ear acupuncture"[Title/Abstract]  **#12**：#10 or #11  **#13**：#9 and #12 | 54 |
| **Web of Science** | **#1**：((((((((((((TS=("Pulmonary Disease, Chronic Obstructive")) OR TS=("Chronic Obstructive Lung Disease")) OR TS=("Chronic Obstructive Pulmonary Diseases")) OR TS=("Chronic Obstructive Pulmonary Disease")) OR TS=("Chronic Obstructive Airway Disease")) OR TS=(COPD)) OR TS=(COAD)) OR TS=("Airflow Obstruction, Chronic")) OR TS=("Airflow Obstructions, Chronic")) OR TS=("Airflow Obstruction, Chronics")) OR TS=("Chronic Airflow Obstructions")) OR TS=("Chronic Airflow Obstruction")) OR TS=("Lung Diseases, Obstructive")  **#2**：((((((((((((TS=("Symptom Flare Up")) OR TS=("recurrent disease")) OR TS=("acute exacerbation")) OR TS=("acute management")) OR TS=("infective exacerbation")) OR TS=("exacerbation")) OR TS=("aggravation")) OR TS=("inpatient")) OR TS=("inpatients")) OR TS=("hospital patient")) OR TS=("hospital admission")) OR TS=("hospitalization")) OR TS=("rehospitalization")  **#3**：#1 and #2  **#4**：(((TS=("acute exacerbation of chronic obstructive pulmonary disease")) OR TS=("AECOPD")) OR TS=("acute exacerbation of copd")) OR TS=("exacerbation of copd")  **#5**：#3 or #4  **#6**：((((((((((((TS=("Acupuncture")) OR TS=("Acupuncture Therapy")) OR TS=("acupuncture points")) OR TS=("body acupuncture")) OR TS=("manual acupuncture")) OR TS=("electroacupuncture")) OR TS=("electro-acupuncture")) OR TS=("warm acupuncture")) OR TS=("ear acupuncture")) OR TS=("Needle")) OR TS=("Needling")) OR TS=("Pricking")) OR TS=("Bloodletting")  **#7**：#5 and #6 | 65 |
| **Cochrane Library** | **#1**: MeSH descriptor: [Pulmonary Disease, Chronic Obstructive] explode all trees  **#2**: (Pulmonary Disease, Chronic Obstructive):ti,ab,kw OR (Chronic Obstructive Pulmonary Diseases):ti,ab,kw OR (Chronic Obstructive Pulmonary Disease):ti,ab,kw OR (Chronic Obstructive Airway Disease):ti,ab,kw OR (COPD):ti,ab,kw OR (COAD):ti,ab,kw OR (Airflow Obstruction, Chronic):ti,ab,kw OR (Airflow Obstructions, Chronic):ti,ab,kw OR (Airflow Obstruction, Chronics):ti,ab,kw OR (Chronic Airflow Obstructions):ti,ab,kw OR (Chronic Airflow Obstruction):ti,ab,kw OR (Lung Diseases, Obstructive):ti,ab,kw  **#3**: #1 OR #2  **#4**: MeSH descriptor: [Symptom Flare Up] explode all trees  **#5**: MeSH descriptor: [Inpatients] explode all trees  **#6**: (recurrent disease):ti,ab,kw OR (acute exacerbation):ti,ab,kw OR (acute management):ti,ab,kw OR (infective exacerbation):ti,ab,kw OR (exacerbation):ti,ab,kw OR (aggravation):ti,ab,kw OR (inpatient):ti,ab,kw OR (hospital patient):ti,ab,kw OR (hospital admission):ti,ab,kw OR (hospitalization):ti,ab,kw OR (rehospitalization):ti,ab,kw  **#7**: #4 OR #5 OR #6  **#8**: #3 AND #7  **#9**: (acute exacerbation of chronic obstructive pulmonary disease):ti,ab,kw OR (AECOPD):ti,ab,kw OR (acute exacerbation of copd):ti,ab,kw OR (exacerbation of copd):ti,ab,kw  **#10**: #8 OR #9  **#11**: MeSH descriptor: [Acupuncture] explode all trees  **#12**: MeSH descriptor: [Acupuncture Therapy] explode all trees  **#13**: (acupuncture points):ti,ab,kw OR (body acupuncture):ti,ab,kw OR (manual acupuncture):ti,ab,kw OR (electroacupuncture):ti,ab,kw OR (warm acupuncture):ti,ab,kw OR (ear acupuncture):ti,ab,kw OR (Needle):ti,ab,kw OR (Needling):ti,ab,kw OR (Pricking):ti,ab,kw OR (Bloodletting):ti,ab,kw  **#14**: #11 OR #12 OR #13  **#15**: #10 AND #14 | 60 |
| **Embase** | **#1**:'chronic obstructive lung disease'/exp OR 'chronic obstructive lung disease'  **#2**:'pulmonary disease, chronic obstructive':ab,ti OR 'pulmonary disease chronic obstructive':ab,ti OR 'chronic obstructive pulmonary diseases':ab,ti OR 'chronic obstructive airway disease':ab,ti OR 'chronic obstructive airway diseases':ab,ti OR 'airflow obstruction chronic':ab,ti OR 'airflow obstructions chronic':ab,ti OR 'airflow obstruction chronics':ab,ti OR 'chronic airflow obstructions':ab,ti OR 'chronic airflow obstruction':ab,ti OR 'lung diseases obstructive':ab,ti  **#3**:#1 OR #2  **#4**:'recurrent disease'/exp OR 'recurrent disease' OR 'hospital patient'/exp OR 'hospital patient'  **#5**:'symptom flare up':ab,ti OR 'recurrent disease':ab,ti OR 'acute exacerbation':ab,ti OR 'acute management':ab,ti OR 'infective exacerbation':ab,ti OR 'exacerbation':ab,ti OR 'aggravation':ab,ti OR 'inpatient':ab,ti OR 'inpatients':ab,ti OR 'hospital admission':ab,ti OR 'hospitalization':ab,ti OR 'inpatient':ab,ti OR 'inpatients':ab,ti OR 'hospital admission':ab,ti OR 'hospitalization':ab,ti OR 'rehospitalization':ab,ti  **#6**:#4 OR #5  **#7**:#3 AND#6  **#8**:'acute exacerbation of chronic obstructive pulmonary disease':ab,ti OR 'AECOPD':ab,ti OR 'acute exacerbation of COPD':ab,ti OR 'exacerbation of COPD':ab,ti  **#9**:#7 OR #8  **#10**:'acupuncture'/exp OR acupuncture  **#11**:'Acupuncture Therapy':ab,ti OR 'acupuncture points':ab,ti OR 'body acupuncture':ab,ti OR 'manual acupuncture':ab,ti OR 'electroacupuncture':ab,ti OR 'electro-acupuncture':ab,ti OR 'warm acupuncture':ab,ti OR 'Needle':ab,ti OR 'Needling':ab,ti OR 'Pricking':ab,ti OR 'Bloodletting':ab,ti OR 'ear acupuncture':ab,ti  **#12**:#10 or #11  **#13**:#9 and #12 | 138 |
| **CINAHL** | **S1** (MH "Pulmonary Disease, Chronic Obstructive")  **S2** MH (MH "Pulmonary Disease, Chronic Obstructive") OR TI ( pulmonary disease chronic obstructive or chronic obstructive lung disease or chronic obstructive pulmonary diseases or COPD or COAD or chronic obstructive airway disease or chronic obstructive airway diseases or airflow obstruction chronic or airflow obstructions chronic or airflow obstruction chronics or chronic airflow obstruction or chronic airflow obstruction or lung diseases obstructive ) OR AB ( pulmonary disease chronic obstructive or chronic obstructive lung disease or chronic obstructive pulmonary diseases or COPD or COAD or chronic obstructive airway disease or chronic obstructive airway diseases or airflow obstruction chronic or airflow obstructions chronic or airflow obstruction chronics or chronic airflow obstruction or chronic airflow obstruction or lung diseases obstructive )  **S3** (MH "Acute Disease")  **S4** MH (MH "Acute Disease") OR TI ( symptom flare up or recurrent disease or acute exacerbation or acute management or infective exacerbation or exacerbation or aggravation ) OR AB ( symptom flare up or recurrent disease or acute exacerbation or acute management or infective exacerbation or exacerbation or aggravation )  **S5** (MH "Inpatients")  **S6** MH (MH "Inpatients") OR TI ( Inpatient or hospital patient or hospital admission or hospitalization or rehospitalization ) OR AB ( Inpatient or hospital patient or hospital admission or hospitalization or rehospitalization )  **S7** S4 OR S6  **S8** S2 AND S7  **S9** TI ( acute exacerbation of chronic obstructive pulmonary disease or AECOPD or acute exacerbation of copd or exacerbation of copd ) AND AB ( acute exacerbation of chronic obstructive pulmonary disease or AECOPD or acute exacerbation of copd or exacerbation of copd )  **S10** s8 or s9  **S11** (MH "Acupuncture")  **S12** MH (MH "Acupuncture") OR TI ( acupuncture therapy or acupuncture points or body acupuncture or manual acupuncture or electroacupuncture or electro-acupuncture or warm acupuncture or Needle or Needling or Pricking or Bloodletting or ear acupuncture ) OR AB ( acupuncture therapy or acupuncture points or body acupuncture or manual acupuncture or electroacupuncture or electro-acupuncture or warm acupuncture or Needle or Needling or Pricking or Bloodletting or ear acupuncture )  **S13** S10 AND S12) | 8 |
| **AMED** | **S1** TI ( pulmonary disease, chronic obstructive or pulmonary disease chronic obstructive or chronic obstructive lung disease or chronic obstructive pulmonary diseases or COPD or COAD or chronic obstructive airway disease or chronic obstructive airway diseases or airflow obstruction chronic or airflow obstructions chronic or airflow obstruction chronics or chronic airflow obstruction or chronic airflow obstruction or lung diseases obstructive ) OR KW ( Pulmonary Disease, Chronic Obstructive or pulmonary disease chronic obstructive or chronic obstructive lung disease or chronic obstructive pulmonary diseases or COPD or COAD or chronic obstructive airway disease or chronic obstructive airway diseases or airflow obstruction chronic or airflow obstructions chronic or airflow obstruction chronics or chronic airflow obstruction or chronic airflow obstruction or lung diseases obstructive ) OR AB ( Pulmonary Disease, Chronic Obstructive or pulmonary disease chronic obstructive or chronic obstructive lung disease or chronic obstructive pulmonary diseases or COPD or COAD or chronic obstructive airway disease or chronic obstructive airway diseases or airflow obstruction chronic or airflow obstructions chronic or airflow obstruction chronics or chronic airflow obstruction or chronic airflow obstruction or lung diseases obstructive )  **S2** TI ( pulmonary disease, chronic obstructive or pulmonary disease chronic obstructive or chronic obstructive lung disease or chronic obstructive pulmonary diseases or COPD or COAD or chronic obstructive airway disease or chronic obstructive airway diseases or airflow obstruction chronic or airflow obstructions chronic or airflow obstruction chronics or chronic airflow obstruction or chronic airflow obstruction or lung diseases obstructive ) OR KW ( Pulmonary Disease, Chronic Obstructive or pulmonary disease chronic obstructive or chronic obstructive lung disease or chronic obstructive pulmonary diseases or COPD or COAD or chronic obstructive airway disease or chronic obstructive airway diseases or airflow obstruction chronic or airflow obstructions chronic or airflow obstruction chronics or chronic airflow obstruction or chronic airflow obstruction or lung diseases obstructive ) OR AB ( Pulmonary Disease, Chronic Obstructive or pulmonary disease chronic obstructive or chronic obstructive lung disease or chronic obstructive pulmonary diseases or COPD or COAD or chronic obstructive airway disease or chronic obstructive airway diseases or airflow obstruction chronic or airflow obstructions chronic or airflow obstruction chronics or chronic airflow obstruction or chronic airflow obstruction or lung diseases obstructive )  **S3 S1 AND S2**  **S4** TI ( acute exacerbation of chronic obstructive pulmonary disease or AECOPD or acute exacerbation of copd or exacerbation of copd ) OR KW ( acute exacerbation of chronic obstructive pulmonary disease or AECOPD or acute exacerbation of copd or exacerbation of copd ) OR AB ( acute exacerbation of chronic obstructive pulmonary disease or AECOPD or acute exacerbation of copd or exacerbation of copd )  **S5 S3** OR **S4**  **S6** TI ( Acupuncture or acupuncture therapy or acupuncture points or body acupuncture or manual acupuncture or electroacupuncture or electro-acupuncture or warm acupuncture or Needle or Needling or Pricking or Bloodletting or ear acupuncture ) OR KW ( Acupuncture or acupuncture therapy or acupuncture points or body acupuncture or manual acupuncture or electroacupuncture or electro-acupuncture or warm acupuncture or Needle or Needling or Pricking or Bloodletting or ear acupuncture ) OR AB ( Acupuncture or acupuncture therapy or acupuncture points or body acupuncture or manual acupuncture or electroacupuncture or electro-acupuncture or warm acupuncture or Needle or Needling or Pricking or Bloodletting or ear acupuncture )  **S7 S5** AND **S6** | 4 |
| **China National Knowledge Infrastructure (CNKI, Chinese Database)** | SU=（慢阻肺急性加重+慢性阻塞性肺病急性加重+慢性阻塞性肺疾病急性加重+慢阻肺急性发作+慢性阻塞性肺病急性发作+慢性阻塞性肺疾病急性发作+AECOPD+COPD急性加重+COPD急性发作）AND SU=(针刺+针刺疗法+针灸+电针+温针+体针+耳针+腹针+经皮电刺激+刺络放血）AND TKA=（随机+对照+临床+RCT） | 146 |
| **Chinese Biomedical Literature Database**  **(CBM, Chinese Database)** | ("随机"[摘要:智能] OR "对照"[摘要:智能] OR "临床"[摘要:智能] OR "RCT"[摘要:智能]) AND ((("针刺疗法"[常用字段:智能] OR "体针"[常用字段:智能] OR "温针"[常用字段:智能] OR "电针"[常用字段:智能] OR "耳针"[常用字段:智能] OR "腹针"[常用字段:智能] OR "经皮电刺激"[常用字段:智能] OR "针灸"[常用字段:智能] OR "刺络放血"[常用字段:智能]) OR ("针刺"[不加权:扩展])) AND (((("慢性阻塞性肺疾病"[常用字段:智能] OR "慢性阻塞肺疾病"[常用字段:智能] OR "慢性气道阻塞性疾病"[常用字段:智能] OR "慢性气流阻塞"[常用字段:智能] OR "慢性气道阻塞"[常用字段:智能] OR "COPD"[常用字段:智能]) OR ("肺疾病, 慢性阻塞性"[不加权:扩展])) AND ("急性加重期"[常用字段:智能] OR "急性发作期"[常用字段:智能] OR "急性加重"[常用字段:智能] OR "急性发作"[常用字段:智能])) OR ("AECOPD"[常用字段:智能]))) | 451 |
| **Chongqing VIP Database**  **(VIP, Chinese Database)** | (M=(慢阻肺急性加重 OR 慢性阻塞性肺病急性加重 OR 慢性阻塞性肺疾病急性加重 OR 慢阻肺急性发作 OR 慢性阻塞性肺病急性发作 OR 慢性阻塞性肺疾病急性发作 OR AECOPD OR COPD急性加重 OR COPD急性发作) OR R=(慢阻肺急性加重 OR 慢性阻塞性肺病急性加重 OR 慢性阻塞性肺疾病急性加重 OR 慢阻肺急性发作 OR 慢性阻塞性肺病急性发作 OR 慢性阻塞性肺疾病急性发作 OR AECOPD OR COPD急性加重 OR COPD急性发作)) AND (M=(针刺 OR 针刺疗法 OR 针灸 OR 电针 OR 温针 OR 体针 OR 耳针 OR 腹针 OR 经皮电刺激 OR 刺络放血) OR R=(针刺 OR 针刺疗法 OR 针灸 OR 电针 OR 温针 OR 体针 OR 耳针 OR 腹针 OR 经皮电刺激 OR 刺络放血)) AND R=(随机 OR 对照 OR 临床 OR RCT) | 401 |
| **Wanfang Database**  **(WF, Chinese Database)** | 主题：（慢阻肺急性加重 OR 慢性阻塞性肺病急性加重 OR 慢性阻塞性肺疾病急性加重 OR 慢阻肺急性发作 OR 慢性阻塞性肺病急性发作 OR 慢性阻塞性肺疾病急性发作 OR AECOPD OR COPD急性加重 OR COPD急性发作）AND 主题：(针刺 OR 针刺疗法 OR 针灸 OR 电针 OR 温针 OR 体针 OR 耳针 OR 腹针 OR 经皮电刺激 OR 刺络放血) AND 摘要：（随机 OR 对照 OR 临床 OR RCT） | 616 |

**Supplement-2:Details of the Literature Search Strategy**
