# Supplementary material for: Efficacy of acupuncture as adjunctive therapy for patients with acute exacerbation of chronic obstructive pulmonary disease: a systematic review and meta-analysis
Source: Front Med (Lausanne). 2025 May 12;12:1513888. doi: 10.3389/fmed.2025.1513888 (PMC12104076; doi:10.3389/fmed.2025.1513888)
Supplement: Supplementary file 3 [file Data_Sheet_3.docx]

| Study ID | N  （T/C） | Age（T/C） | Acupuncture Details | Acupoints | Manipulation | Treatment | Control | Outcomes |
| --- | --- | --- | --- | --- | --- | --- | --- | --- |
| Guan,W 2016 | 33/34 | T65.17±6.26  C66.53±6.89 | 30min/time;  14d | RN12、RN10、RN6、RN4、ST24、ST25、SP15、GB26 | rotating method | abdominal acupuncture  +CWMT | CWMT | ①②③⑤⑥⑦⑧ |
| ONCU,E 2016 | 35/35 |  | 25min/time;  20d | EX-B1、LU-7 |  | TENS  +CWMT | CWMT | ④⑩ |
| Yang,TH 2022 | 31/30 | T76.71±7.84  C77.77±5.74 | 30min/time;  14d | RN12、RN10、RN6、RN4 | rotating method or/and （Lifting and thrusting method） | abdominal acupuncture  +CWMT | CWMT | ⑤⑥⑦⑪ |
| Xu,YG 2007(1) | 20/20 | T65.0  C66.0 | 4h/time;  28d | RN17 |  | Heavy felting needle+CWMT | CWMT | ⑪ |
| Xu,YG 2007(2) | 20/20 | T63.5  C66.0 | 30min/time;  28d | RN17 |  | filiform needle+CWMT | CWMT | ⑪ |
| Zhan,WX 2013(1) | 30/30 |  | 3h/time;  10d | RN19、RN17 |  | Heavy felting needle+CWMT | CWMT | ②⑧ |
| Zhan,WX 2013(2) | 30/30 |  | 3h/time;  10d | RN19、RN17 |  | filiform needle+CWMT | CWMT | ②⑧ |
| Wen,Q 2011 | 20/20 | T69.70±8.09  C66.90±6.71 | 40min/time;  10d | Ex-B1 |  | TENS  +CWMT | CWMT | ③ |
| Zhou,X 2016 | 30/30 | T70.34±8.22  C71.24±6.31 | 5h/time;  14d | EX-B1、RN17、BL43、Stimulating pain point |  | Fu'sAcupuncture+CWMT | CWMT | ①②③④⑤⑥ |
| Zhang,YQ 2020 | 31/30 | T59.31±7.56  C64.15±8.98 | 5h/time;  3d | RN17、BL12（bilateral）、BL13（bilateral） |  | Fu'sAcupuncture+CWMT | CWMT | ① |
| Zhang,Z 2020 | 50/50 | T61.2±0.5  C63.1±0.3 | 30min/time;  14d | RN12、RN10、RN6、RN4 | rotating method | abdominal acupuncture  +CWMT | CWMT | ① |
| Wang,Y 2020 | 50/50 | T62.17±4.15  C64.44±4.33 | 15min/time;  14d | RN12、RN10、RN4、GB26、ST25、SP15 | rotating method | abdominal acupuncture  +CWMT | CWMT | ①②⑤⑥⑦ |
| Cheng,YY 2017 | 34/34 | 65.3±5.32  64.9±5.72 | 30min/time;  11d | RN12、RN10、RN6、RN4、ST24、ST25、SP15、GB26 |  | abdominal acupuncture  +CWMT | CWMT | ①②③⑤⑥⑦⑧ |
| Gui,K 2023 | 126/125 | 48.73±5.37  49.38±5.42 | 30min/time/  14d | ST40、BL13、LU9、ST36、LU1、SP9 | rotating method and （Lifting and thrusting method） | acupuncture  +CWMT | CWMT | ②③④⑨⑩ |
| Yuan,SC 2021 | 37/37 | 78  83 | 30min/time;  10d | Ex-B1、BL13、RN17、RN15、RN12、LU9、ST36、ST40 | rotating method and （Lifting and thrusting method） | acupuncture  +CWMT | CWMT | ①⑤⑥ |
| Shan,ZL 2020 | 23/24 | 59.52±12.34  60.96±11.59 | 10min/time;  5d | C016、C016、TF4、AT4 |  | ear-acupuncture+CWMT | CWMT | ①⑧⑨ |
| Yuwen,DX 2022 | 20/20 | 71.90±8.80  71.60±8.06 | 45min/time;  3d | A circle centered on DU20 |  | Scalp acupuncture  +CWMT | CWMT | ① |
| Gao,Y 2014 | 31/31 | 67.5  68.2 | 30min/time;  22d | EX-B1 |  | Electroacupuncture+CWMT | CWMT | ②⑧⑨⑩ |
| Zhou,C 2021 | 29/29 | 71.59±5.985  68.69±7.536 | 24-48h/time;  10d | BL18、BL13、LR3、LU9 |  | intradermal needling+CWMT | CWMT | ①⑨ |
| Guo,JQ 2017 | 30/30 | 71.2±7.35  71.8±9.5 | 25min/time;  14d | LU7、LU5、ST40、LU9、KI3、ST36、BL23、BL13、BL20、BL21、BL25、RN12 | Acupuncture Reducing and Reinforcing Method | acupuncture  +CWMT | CWMT | ②③④⑤⑥⑦ |
| Ji,YH 2017 | 31/30 | 56.94±6.557  55.63±6.970 | 30min/time;  14d | RN17、RN12、PC7、BL13、GB20、SJ6、BL12 | rotating method and （Lifting and thrusting method） | acupuncture  +CWMT | CWMT | ①②③⑩ |
| Chen,ZY 2013 | 29/28 | 71.72±6.73  73.14±6.33 | 40min/time;  14d | DU20、EX-HN1、ST9、 |  | acupuncture  +CWMT | CWMT | ①③⑨ |
| Peng,YN 2023 | 58/58 | 68.43±9.188  68.53±8.415 | 30min/time;  7d | BL12、BL13、DU14 | rotating method | acupuncture  +CWMT | CWMT | ④⑧ |
| Xie,F 2019 | 30/29 | 64±9  65±8 | 30min/time;  14d | ST40、BL13、LU9、ST36、LU1、SP9 | rotating method | Warm acupuncture+CWMT | CWMT | ③④⑨ |
| Zhang,HD 2022 | 39/39 |  | 30min/time;  14d |  | Acupuncture Reducing and Reinforcing Method | acupuncture  +CWMT | CWMT | ⑪ |
| Huang,B 2023 | 30/30 | 67.77±3.65  68.97±3.94 | 40min/time;  14d | LU1、LU9、RN12、RN4、ST36、KI3、GB12、BL23、BL13、BL20 | Acupuncture Reducing Method | acupuncture  +CWMT | CWMT | ③④⑧ |
| Zhang,YM 2013 | 30/30 | 71.47±7.48  69.76±7.21 | 30min/time;  14d | BL13、SJ6 | rotating method and （Lifting and thrusting method） | acupuncture  +CWMT | CWMT | ①③ |
| Mao,LN 2019 | 20/23 | 67.75±8.873  67.30±8.374 | 30min/time;  7d | ST36 | rotating method | acupuncture  +CWMT | CWMT | ⑤⑥ |
| Chen,B 2023 | 42/42 | 59.31±7.56  64.15±8.98 | 30min/time;  14d | GB26、SP15、ST25、EX-B1、RN17、RN6、RN4、ST36、SP6 | rotating method | acupuncture  +CWMT | CWMT | ①②③⑧⑩ |
| Yan,W 2024 | 50/50 | T61.91±8.55  C62.01±8.91 | 30min/time;  14d | RN17、LU9、KI3、BL20、BL20、ST25、SP6、BL13 | rotating method and （Lifting and thrusting method） | acupuncture  +CWMT | CWMT | ①④ |
| Wang,F 2025 | 35/35 | T63.97±8.61  C62.36±8.69 | 30min/time;  7d | RN17、LU9、BL20、BL13、ST40、ST36、DU14、EX-B1、LU1 |  | TENS  +CWMT | CWMT | ①③④ |
| Long,Y 2024 | 30/30 | T62.79±7.85  C62.36±7.29 | 30min/time;  7d | RN17、EX-B1、ST40、BL13、LU1、RN22、LU6、PC6 | rotating method | acupuncture  +CWMT | CWMT | ①③④⑥⑦⑩ |
| Yang,DL 2024 | 41/41 | T56.32±3.78  C56.41±3.94 | 30min/time;  14d | RN17、ST40、BL43 | Lifting and thrusting method | acupuncture  +CWMT | CWMT | ① |

|  |
| --- |

①Efficiency ②FEV_1_% ③FEV_1_/FVC ④FEV_1_ ⑤PaO_2_ ⑥PaCO_2_ ⑦SaO_2_ ⑧6WMT ⑨CAT ⑩mMRC ⑪success rate of weaning

Note: T, Treatment group; C, Control group; F, female; M, male; CWMT, Conventional Western Medicine Treatment; TENS, Transcutaneous Electrical Nerve Stimulation

**Supplementary material-3.Detailed features of the included literature**
