# Supplementary material for: Efficacy of acupuncture as adjunctive therapy for patients with acute exacerbation of chronic obstructive pulmonary disease: a systematic review and meta-analysis
Source: Front Med (Lausanne). 2025 May 12;12:1513888. doi: 10.3389/fmed.2025.1513888 (PMC12104076; doi:10.3389/fmed.2025.1513888)
Supplement: Supplementary file 4 [file Data_Sheet_4.docx]

**Author(s): Guofeng Li**
**Date:** 2025-03-07
**Question:** routine treatments plus acupuncture vs routine treatments for AECOPD
**Settings:**
**Bibliography:** . Acupuncture for AECOPD. Cochrane Database of Systematic Reviews [Year], Issue [Issue].

| **Quality assessment** | | | | | | | **No of patients** | | **Effect** | | **Quality** | **Importance** |
| --- | --- | --- | --- | --- | --- | --- | --- | --- | --- | --- | --- | --- |
|  |  |  |  |  |  |  |  |  |  |  |  |  |
| **No of studies** | **Design** | **Risk of bias** | **Inconsistency** | **Indirectness** | **Imprecision** | **Other considerations** | **Routine treatments plus acupuncture** | **Routine treatments** | **Relative (95% CI)** | **Absolute** |  |  |
| **efficiency** | | | | | | | | | | | | |
| 18 | randomised trials | serious^1^ | serious^2^ | no serious indirectness | no serious imprecision | reporting bias^3^ | 586/625  (93.8%) | 480/624  (76.9%) | RR 1.22 (1.16 to 1.28) | 169 more per 1000 (from 123 more to 215 more) | ⊕OOO VERY LOW | CRITICAL |
|  |  |  |  |  |  |  |  | 74.1% |  | 163 more per 1000 (from 119 more to 207 more) |  |  |
| **FEV1% (Better indicated by lower values)** | | | | | | | | | | | | |
| 11 | randomised trials | no serious risk of bias | serious^2^ | no serious indirectness | no serious imprecision | none | 467 | 466 | - | MD 5.67 higher (2.97 to 8.37 higher) | ⊕⊕⊕O MODERATE | CRITICAL |
| **FEV1/FVC (Better indicated by lower values)** | | | | | | | | | | | | |
| 14 | randomised trials | serious^1^ | serious^2^ | no serious indirectness | no serious imprecision | none | 530 | 527 | - | MD 4.44 higher (1.86 to 7.03 higher) | ⊕⊕OO LOW | CRITICAL |
| **FEV1 (Better indicated by lower values)** | | | | | | | | | | | | |
| 10 | randomised trials | serious^1^ | serious^2^ | no serious indirectness | no serious imprecision | none | 123 | 123 | - | MD 0.3 higher (0.18 to 0.43 higher) | ⊕⊕OO LOW | CRITICAL |
| **PaO2 (Better indicated by lower values)** | | | | | | | | | | | | |
| 8 | randomised trials | serious^4^ | serious^2^ | no serious indirectness | no serious imprecision | none | 265 | 268 | - | MD 5.56 higher (2.91 to 8.22 higher) | ⊕⊕OO LOW | CRITICAL |
| **PaCO2 (Better indicated by lower values)** | | | | | | | | | | | | |
| 9 | randomised trials | serious^4^ | serious^2^ | no serious indirectness | no serious imprecision | none | 295 | 298 | - | MD 3.3 lower (5.8 to 0.8 lower) | ⊕⊕OO LOW | CRITICAL |
| **SaO2 (Better indicated by lower values)** | | | | | | | | | | | | |
| 6 | randomised trials | serious^4^ | no serious inconsistency | no serious indirectness | no serious imprecision | none | 208 | 208 | - | MD 8.19 higher (7.15 to 9.23 higher) | ⊕⊕⊕O MODERATE | CRITICAL |
| **6MWT (Better indicated by lower values)** | | | | | | | | | | | | |
| 9 | randomised trials | serious^1^ | serious^2^ | no serious indirectness | no serious imprecision | none | 311 | 313 | - | MD 52.85 higher (31.99 to 73.7 higher) | ⊕⊕OO LOW |  |
| **CAT (Better indicated by lower values)** | | | | | | | | | | | | |
| 6 | randomised trials | serious^4^ | serious^2^ | no serious indirectness | no serious imprecision | none | 268 | 266 | - | MD 2.94 lower (4.27 to 1.61 lower) | ⊕⊕OO LOW | CRITICAL |
| **mMRC (Better indicated by lower values)** | | | | | | | | | | | | |
| 6 | randomised trials | serious^1^ | serious^2^ | no serious indirectness | no serious imprecision | none | 295 | 294 | - | MD 0.33 lower (0.47 to 0.2 lower) | ⊕⊕OO LOW | CRITICAL |
| **Success rate of weaning** | | | | | | | | | | | | |
| 4 | randomised trials | serious^4^ | no serious inconsistency | no serious indirectness | no serious imprecision | none | 76/110  (69.1%) | 63/109  (57.8%) | RR 1.18 (0.95 to 1.48) | 104 more per 1000 (from 29 fewer to 277 more) | ⊕⊕⊕O MODERATE |  |
|  |  |  |  |  |  |  |  | 50% |  | 90 more per 1000 (from 25 fewer to 240 more) |  |  |

^1^ The blind method has a high risk of bias
^2^ High heterogeneity.
^3^ High publication bias
^4^ Randomization has a high risk of bias
